# The CD63-Syntenin-1 Complex Controls Post-Endocytic Trafficking of Oncogenic Human Papillomaviruses

**DOI:** 10.1038/srep32337

**Published:** 2016-08-31

**Authors:** Linda Gräßel, Laura Aline Fast, Konstanze D. Scheffer, Fatima Boukhallouk, Gilles A. Spoden, Stefan Tenzer, Klaus Boller, Ruzica Bago, Sundaresan Rajesh, Michael Overduin, Fedor Berditchevski, Luise Florin

**Affiliations:** 1 Department of Medical Microbiology and Hygiene, University Medical Centre of the Johannes Gutenberg University, Mainz, Germany; 2 Department of Immunology, University Medical Center of the Johannes Gutenberg University, Mainz, Germany; 3 Paul Ehrlich Institute, Langen, Germany; 4 School of Cancer Sciences and Department of Pathology, The University of Birmingham, Birmingham, United Kingdom; 5 Department of Biochemistry, Faculty of Medicine and Dentistry, University of Alberta, Edmonton, Canada

## Abstract

Human papillomaviruses enter host cells via a clathrin-independent endocytic pathway involving tetraspanin proteins. However, post-endocytic trafficking required for virus capsid disassembly remains unclear. Here we demonstrate that the early trafficking pathway of internalised HPV particles involves tetraspanin CD63, syntenin-1 and ESCRT-associated adaptor protein ALIX. Following internalisation, viral particles are found in CD63-positive endosomes recruiting syntenin-1, a CD63-interacting adaptor protein. Electron microscopy and immunofluorescence experiments indicate that the CD63-syntenin-1 complex controls delivery of internalised viral particles to multivesicular endosomes. Accordingly, infectivity of high-risk HPV types 16, 18 and 31 as well as disassembly and post-uncoating processing of viral particles was markedly suppressed in CD63 or syntenin-1 depleted cells. Our analyses also present the syntenin-1 interacting protein ALIX as critical for HPV infection and CD63-syntenin-1-ALIX complex formation as a prerequisite for intracellular transport enabling viral capsid disassembly. Thus, our results identify the CD63-syntenin-1-ALIX complex as a key regulatory component in post-endocytic HPV trafficking.

Human papillomaviruses (HPV) are DNA tumour viruses that inflict a significant burden of disease on the population. The clinical manifestations caused by HPV range from anogenital warts and laryngeal papillomas to cancers at anogenital sites, on the skin and in the oropharynx^1^. The HPV capsid is composed of the major capsid protein L1 and the minor capsid protein L2. L1 and L2 are key players in early events of infection, such as virus binding at the plasma membrane, cellular entry, and transport of the viral DNA into the nucleus^2^,^3^.

Recent research has led to a more detailed picture of HPV entry. The initial events of HPV infection including binding to target cells and transition towards the entry points have been characterized^2^,^4^,^5^,^6^. Specifically, it has been proposed that infectious particles gain access to mitotically active basal cells of skin and mucosa through micro-abrasions in the epithelium by attachment to heparan sulphate proteoglycans^7^,^8^,^9^,^10^ or to distinct components of the extracellular matrix^11^,^12^ as initial binding sites^5^. Subsequent conformational changes of the capsid and cleavage of both capsid proteins occur on the cell surface^13^,^14^ supporting infection and transfer to the entry receptor complex^4^,^9^,^13^,^14^,^15^,^16^. L2 interacts with the annexin A2 heterotetramer^17^,^18^ and the virion associates with additional proteins such as α6 integrins (α6β1/β4)^19^,^20^, growth factor receptors^21^, and tetraspanins^22^,^23^,^24^,^25^. However, the early post-entry endocytic pathway of HPV remains poorly defined. Following internalisation, viral particles are transported to multivesicular endosomes (MVEs)^26^, where combined with acidification, HPV capsid undergoes disassembly/uncoating, a prerequisite for successful infection^27^,^28^,^29^,^30^,^31^. It has been recently reported that the ESCRT-1 subunit, TSG101, directly interacts with the minor capsid protein L2 and is required for papillomavirus infection^32^. These findings support the notion that the delivery of viral particles to MVEs is an essential step in HPV infection which precedes the retrograde pathway from the endosomal system to the Golgi/ER compartment^30^,^33^,^34^ during its journey into the nucleus^35^,^36^,^37^,^38^,^39^. However, cellular proteins that regulate the passage of HPV from the plasma membrane through endosomal compartments are unknown.

Our previous studies have shown that the secondary receptor complex is located within tetraspanin-enriched microdomains (TEMs or TERMs) which are likely to function as entry platforms for various papillomaviruses^22^,^23^,^24^,^25^ thus enabling virus internalisation via an endocytic mechanism independent of clathrin, caveolin and, dynamin^22^,^24^,^26^,^40^. Furthermore, we established that association of viral particles with the tetraspanin CD151-integrin complexes and integration of these complexes to TERMs are required for virus endocytosis^23^. Given that several tetraspanin proteins include well-defined endocytic targeting sequences^41^, we proposed that they may be responsible for post-endocytic trafficking of HPV^6^,^22^,^23^,^24^.

Here, we investigated the role of tetraspanin CD63 and its cytoplasmic partner syntenin-1 in HPV infection. Both CD63 and syntenin-1 are abundant in MVEs and known to regulate endocytic trafficking of associated cargos^41^,^42^,^43^. We demonstrate that infectivity of HPV is dependent on the CD63-syntenin-1 complex. Importantly, we established that this complex controls post-endocytosis trafficking of the virus to MVEs through a mechanism involving ALG-2-interacting protein X (ALIX), a recently described partner of syntenin-1^44^ which regulates MVE biogenesis. Together, these results identify the CD63-syntenin-1-ALIX complex as a key component of a novel trafficking pathway which regulates post-endocytic sorting of HPV.

## Results

### Tetraspanin CD63 controls infectivity of HPV

**Figure 1:**
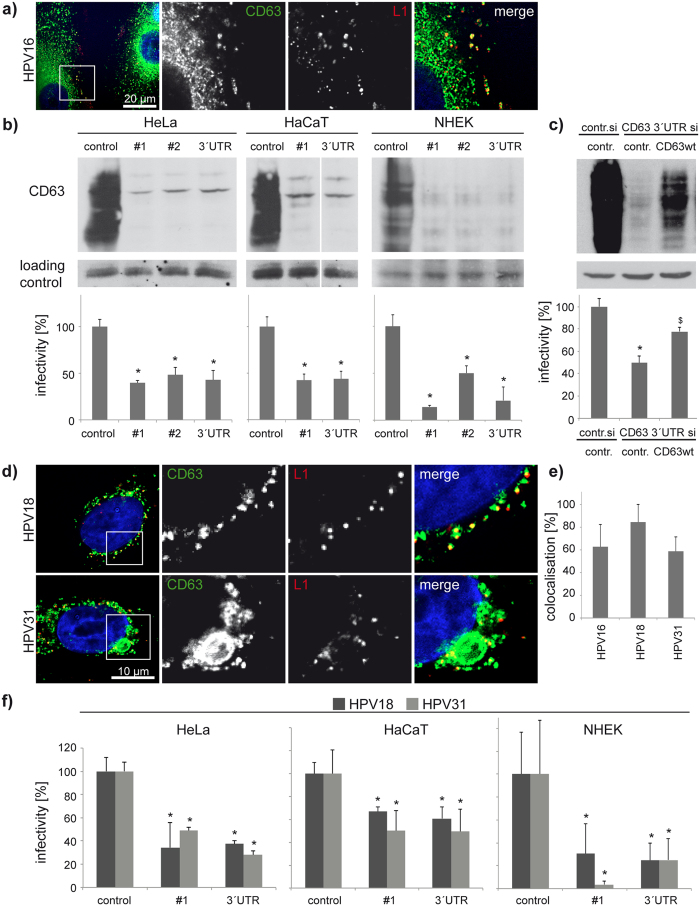
CD63 is required for HPV16, -18, and -31 infections. (**a**) HPV16 is internalised into CD63-positive endosomal compartments in primary keratinocytes (NHEK). NHEK cells were infected with HPV16 for 7 hours and stained with CD63 monoclonal antibody (mAb) and L1-polyclonal antibody (pAb) K75. Shown are representative pictures of endogenous CD63 (green) and HPV16 L1 (red). (**b**) CD63 knockdown correlates with reduced infectivity. Upper panels show efficiency of CD63 knockdown in HeLa, HaCaT and NHEK cells by Western blotting. Bottom panels show HPV16 pseudovirus (PsV) infection assay of HeLa, HaCaT and NHEK cells after CD63 siRNA treatment. Infectivity was measured by luciferase activity and normalized by LDH measurements. Infection rate of control siRNA was set to 100%. (**c**) HPV16 infectivity can be restored after reexpression in CD63 depleted cells. HeLa cells were treated with CD63 siRNAs, transfected with CD63-expression or control plasmid and infected with HPV16 PsV. Infectivity of HPV16 PsV was analysed as above. Upper panels show the efficiency of knockdown and reexpression. (**d**) HPV18 and -31 are internalised into CD63-positive endosomal compartments. NHEK cells were infected with HPV18 or HPV31 PsV and analysed as in (**a**). (**e**) Quantification of L1 colocalisation with CD63 was performed by analysis of at least 12 images (3–5 cells per image) using Colocalisation Software 4.7 (Zeiss). Shown is the proportion of CD63-colocalising L1 pixels relative to the total amount of L1 pixels. (**f**) HeLa, HaCaT, and NHEK cells were treated as in (**b**) and HPV18 and HPV31 PsV infectivity after CD63 knockdown was analysed as above. *P < 0.05 significant decrease compared to control, ^$^*P* < 0.05 significant increase compared to cells transfected with CD63 3′UTR siRNA and control plasmid.

We have previously reported that HPV16 pseudoviruses (PsV) colocalise with tetraspanin CD63 on the plasma membrane and in endosomes of infected HeLa cells^22^. Similarly, HPV16 L1 colocalises with CD63 in primary keratinocytes (NHEK) (Fig. 1a). To investigate whether CD63 plays a role in HPV infection, we performed a series of infection assays using various keratinocyte cell models depleted of CD63. These experiments demonstrated that CD63 knockdown with different siRNAs decreased HPV16 infection of HeLa, HaCaT and primary keratinocytes by >50% (Fig. 1b). Control experiments showed that CD63 depletion does not affect luciferase gene expression (Supplementary Fig. S1). Importantly, reexpression of CD63 in siRNA-depleted HeLa cells restored viral infectivity, excluding possible off-target effects from siRNA-based knockdowns (Fig. 1c). To extend these observations we analysed the role of CD63 in infection of two other oncogenic HPV types: HPV18 and HPV31. Immunofluorescence staining demonstrated that HPV18 and HPV31 L1 proteins also strongly colocalise with CD63 suggesting that these viruses were internalised into CD63-positive endosomes (Fig. 1d). Detailed quantification revealed that ~60% of HPV 16, 18, and 31 L1-positive endosomes were labelled with anti-CD63 mAb (Fig. 1e). Moreover, depletion of CD63 inhibited HPV18 and HPV31 infection in all cell lines tested (Fig. 1f). These data demonstrate that CD63 plays a general role in infection of epithelial cells with various human papillomaviruses.

### CD63 regulates post-endocytic steps in HPV infection

**Figure 2:**
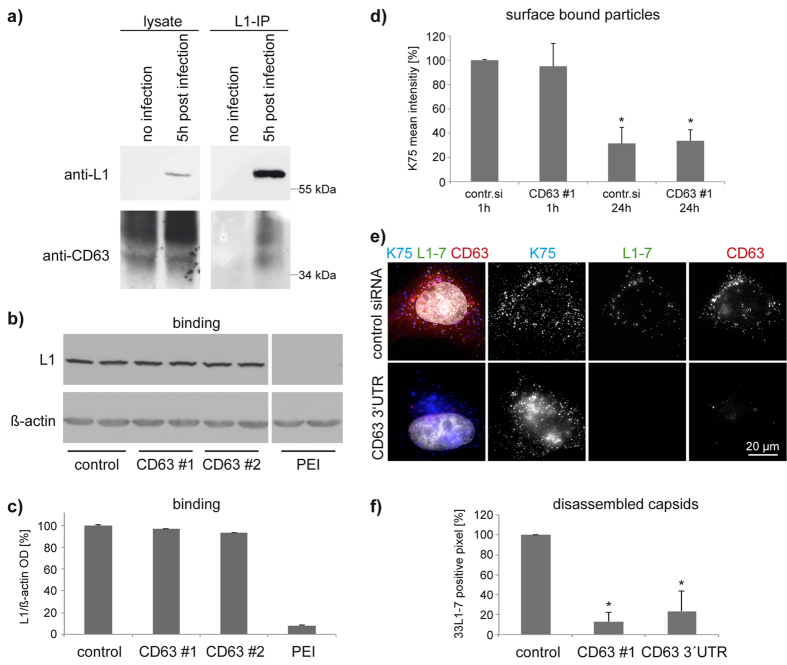
CD63 associates with L1 and is involved in HPV16 post-uncoating processing but not in cell surface binding or virus endocytosis. (**a**) CD63 is coprecipitated by L1 pAb K75 immunoprecipitation (IP) from lysates of HPV16 PsV infected HeLa cells. Expressed (left) and precipitated proteins (right) were detected by Western blotting using L1 mAb 321-F (upper panels) and mAb CD63 (lower panels). (**b–d**) CD63 knockdown does not affect HPV16 cell binding. (**b**) HeLa cells were pretreated with CD63 or control siRNA and incubated with HPV16 PsV. Cell-bound PsV were detected in cell lysates by Western blotting (WB) using L1 mAb 312F. Polyethylenimine (PEI) treated cells were used as control for binding deficiency^75^. (**c**) Quantification of WB bands shows HPV16 cell surface binding independent of CD63. (**d**) PsV binding and endocytosis is unaffected by CD63 knockdown. HeLa cells were pretreated as in (**b**). Cells were incubated with HPV16 PsV as indicated. The amount of surface associated PsV was assessed by flow cytometry using L1 pAb K75. The difference between the amount of surface bound PsV at 1 h and 24 h serves as a measure for endocytosis. Mean fluorescence intensity of cells incubated with PsV for 1 h was adjusted to 100%. (**e,f**) L1-7 reactivity is reduced after CD63 knockdown. Representative pictures of immunofluorescence with HPV16 L1 pAb K75 (blue), mAb 33L1-7 (green) and CD63 mAb (red) are shown in (**e**). Hela cells were treated with siRNAs as in (**b**) and infected for 7 hours. K75 Ab recognises bound and internalized virus particles. 33L1-7 Ab recognises HPV16 epitope only accessible after capsid disassembly. (**f**) Shows quantification of disassembled viral capsids performed by analysis of L1-7 positive pixels of at least 15 images using ImageJ-script. Shown are the results of three independent experiments using CD63 siRNAs normalized to control siRNA treated cells. *P < 0.05 compared to control.

To investigate the involvement of CD63 in HPV infection in more detail we first examined the interaction of HPV16 PsV with CD63. L1-specific coimmunoprecipitation assay confirmed that HPV16 is physically linked to the CD63-containing protein complex (Fig. 2a). Next, we analysed the functional requirement of CD63 for virus cell binding, endocytosis, and post-entry processing of HPV16 PsV in CD63-depleted cells. As shown by Western blotting (Fig. 2b,c) and flow cytometry (Figs 1h and 2d), binding of HPV16 PsV to the surface of HeLa/CD63(-) cells was similar to that of control cells. Furthermore, flow cytometry experiments demonstrated that depletion of CD63 had no apparent effect on internalisation of HPV16: staining intensity of polyclonal anti-L1 antibody K75, which detects surface bound PsV, was comparable in control and CD63-depleted cells 24 hours after infection (Fig. 2d). To investigate the effect of CD63 depletion on virus post-uncoating processing, we examined the amount of disassembled capsids in endosomes using the disassembly-specific L1 antibody 33L1-7 (short L1-7)^22^,^29^,^45^ which detects L1 capsomeres, unfolded L1^46^, and also post-disassembly degraded L1^14^. This antibody binds a linear epitope (residues 303–313) that is inaccessible in intact viral particles^46^,^47^ and recognizes L1 protein after capsid disassembly^22^. Notably, the number of endosomes positive for the L1-7 epitope was dramatically decreased in CD63-depleted cells (Fig. 2e,f), while staining with the polyclonal L1-antibody K75 was unaffected (Fig. 2d,e). These findings indicate that CD63 is not involved in HPV cell binding or endocytosis. Instead, CD63 appears to control a step in HPV infection, which follows endocytosis but precedes virus uncoating and post-uncoating processes.

To examine CD63-dependent trafficking of HPV16 through the endosomal pathway, we also analysed L1 cleavage products. It was shown that most of L1 cleavages occur in acidified endosomes generating products of degradation^14^. In these experiments we found that CD63 depletion reduced the generation of lower molecular weight L1 cleavage products (15–30 kDa) (Supplementary Fig. S1). In further control experiments, we analysed whether depletion of CD63 would affect trafficking of transferrin or epidermal growth factor (EGF). Transferrin is a bona fide cargo for the clathrin-dependent endocytic pathway^48^ and EGF is internalized by endocytosis followed by trafficking through the various endocytic compartments including multivesicular endosomes (MVEs) for degradation^49^. CD63 did not colocalise with transferrin under steady state conditions and its depletion did not affect the amount or intracellular distribution of endocytosed transferrin (Supplementary Fig. S2). Similarly, CD63 knockdown had no effect on EGF cell entry or trafficking of the growth factor to MVEs (marked with anti-LBPA mAb, Supplementary Fig. S3). These results emphasise that CD63 specifically acts on virus-containing endosomes.

### CD63 regulates HPV infection via syntenin-1

**Figure 3:**
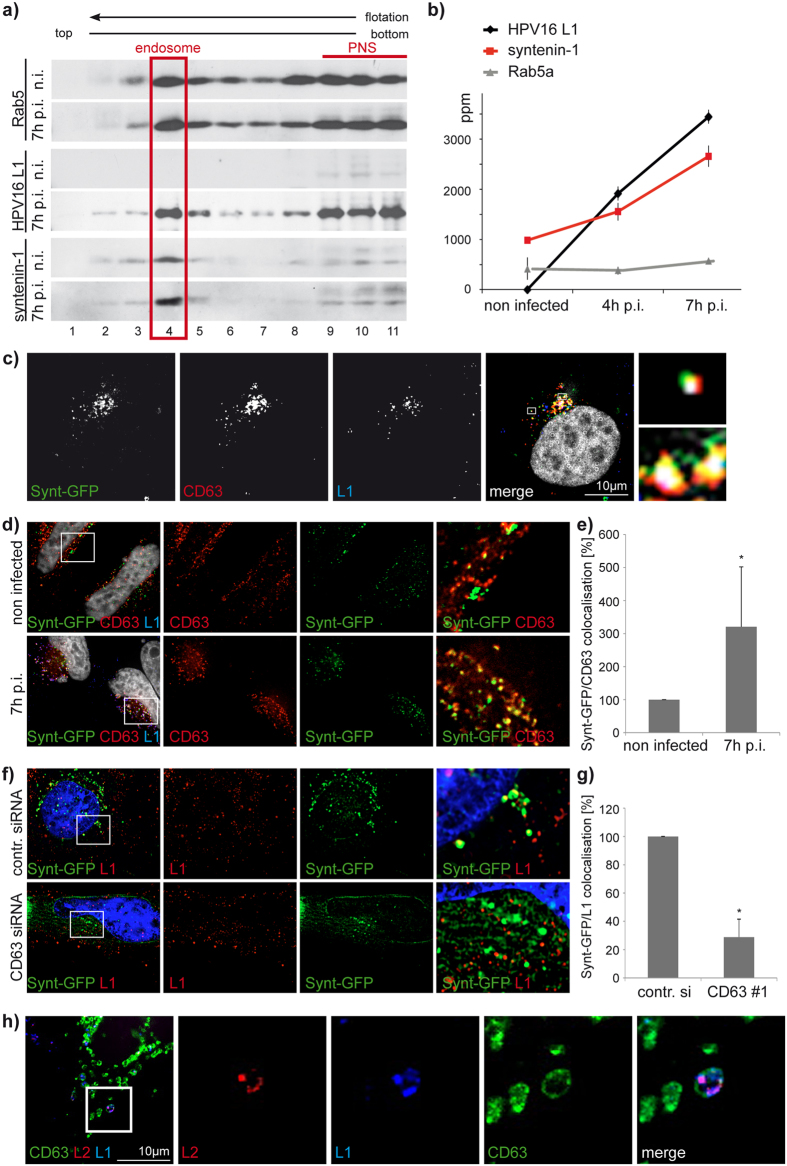
Recruitment of syntenin-1 to endosomes after HPV16 infection. (**a,b**) Endosomal syntenin-1 increased according to HPV incubation time. Western blot (**a**) and label-free quantitative mass spectrometry (**b**) of L1, syntenin-1 and Rab5/Rab5a in HeLa cells infected with HPV16 PsV for the indicated time points. Rab5 marks early endosomes and serves as loading control. Fraction 4 in the Western blot shows endosomal fractions used for mass spectrometry. Fractions 9 to 11 represent post-nuclear supernatant (PNS) rich in soluble forms of Rab5. (**c**–**e**) Syntenin-GFP expressing HeLa cells show significantly increased colocalisation of CD63 and L1 at 7 hours post infection (7h p.i.). (**c**,**d**) Representative pictures show immunofluorescence of L1 (blue), CD63 (red), and syntenin-GFP (green) expressing HeLa cells. (**e**) Quantification of syntenin-GFP-colocalising CD63 pixels was performed by analysis of at least 10 images (3-5 cells per image) using Colocalisation Software 4.7 (Zeiss). *P < 0.05 compared to control. (**f,g**) Syntenin-1 recruitment to HPV L1 positive endosomes is significantly decreased after CD63 depletion. (**f**) Shows representative pictures of syntenin-GFP (green) and L1 (red) after 7 h infection in control or CD63 siRNA treated HeLa cells. (**g**) Quantification of syntenin-GFP-colocalising L1 pixels was performed by analysis of at least 20 images (3-5 cells per image) using Colocalisation Software 4.7 (Zeiss). *P < 0.05 compared to control. (**h**) CD63 positive endosomes contain both L1 and L2. Representative pictures show HeLa cells transfected with CD63-GFP expression plasmid and stained with pAb K75 and mAb L2-1. CD63-GFP is shown in green, L2 in red, L1 in blue and the nucleus in grey.

We have previously reported that CD63 directly interacts with the PDZ domain-containing adaptor protein syntenin-1, which is known to control various aspects of endocytic trafficking including trafficking of CD63^50^. Therefore, we investigated whether syntenin-1 is involved in CD63-dependent steps of HPV infection. Western blot analysis showed that syntenin-1 together with endosomal marker Rab5 and the HPV L1 was detectable in specific sucrose density fractions of endosomal preparations of infected HeLa cells (Fig. 3a). In addition, Western blot (Fig. 3a) and quantitative mass spectrometry (Fig. 3b) of endosomal preparations revealed that syntenin-1 levels steadily increased on HPV-containing endosomes when assessed at four and seven hours post-infection (Fig. 3b and Supplementary Fig. S4). By contrast, endosomal distribution of CD63 was not affected (data not shown), suggesting the interaction of virus with CD63 induces recruitment of syntenin-1 to endosomal membranes. Supporting these findings, immunofluorescence experiments demonstrated colocalisation of CD63, syntenin-1 and HPV16 L1 protein in HPV16-infected cells (Fig. 3c). Moreover, colocalisation of syntenin-1 and CD63 was significantly increased after 7 hours HPV infection when compared to uninfected controls (Fig. 3d,e) and, conversely, was reduced in CD63 depleted cells compared to control siRNA treated cells (Fig. 3f,g) further emphasizing that CD63 and syntenin-1 interact at HPV-containing endosomes. These endosomes also contain the minor capsid protein L2 (Fig. 3h) suggesting syntenin-1 association takes place prior to capsid disassembly and dissociation of the capsid proteins.

**Figure 4:**
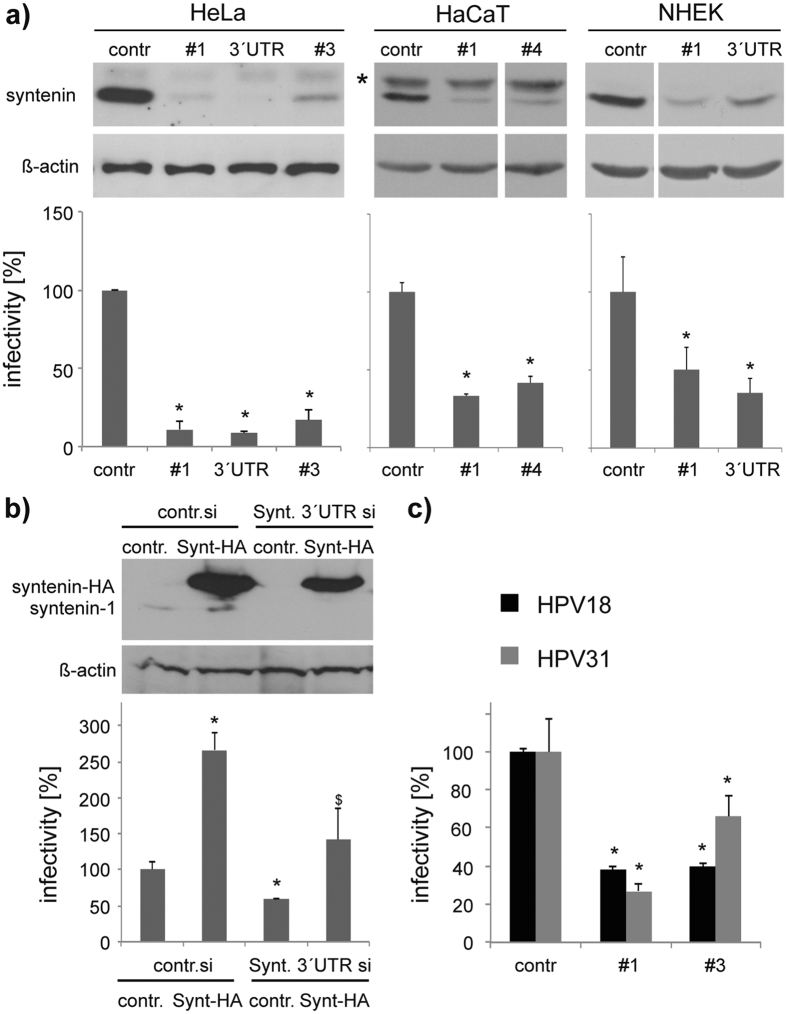
Syntenin-1 is required for papillomavirus infection. (**a**) Syntenin-1 knockdown correlates with reduced HPV16 infectivity. Upper panels show efficiency of syntenin-1 knockdown in HeLa, HaCaT, and NHEK cells (*non-specific band). Bottom panels show correlating HPV16 infection assay in HeLa, HaCaT and NHEK cells after syntenin-1 or control siRNA treatment. Infectivity was measured as in Fig. 1b. (**b**) HPV16 infectivity can be restored after syntenin-1 reexpression. HeLa cells were treated with syntenin-1 siRNAs, transfected with control or syntenin-1-expression plasmid and then infected with HPV16 PsV. Infectivity of HPV16 PsV was analysed as above. Upper panels show the efficiency of syntenin-1 knockdown and reexpression. *P < 0.05 significant decrease compared to control, ^$^*P* < 0.05 significant increase compared to cells transfected with syntenin 3′UTR siRNA and control plasmid. (**c**) Syntenin-1 knockdown correlates with reduced HPV18 and HPV31 infectivity. HeLa cells were treated as in (**a**) and infectivity of HPV18 or HPV31 PsV was analysed as above.

While knockdown of syntenin-1 decreased infectivity of HPV16 PsV in HeLa, HaCaT and NHEK cells (Fig. 4a, control in Supplementary Fig. S1), overexpression of the protein increased viral infectivity (Fig. 4b). The inhibitory phenotype of the knockdown could be reversed when syntenin-1 was reexpressed in siRNA-depleted cells, excluding possible off-target effects and indicating that syntenin-1 plays a key role in HPV infection. Similarly, infectivity of HPV18 and HPV31 PsV was also decreased in syntenin-1-depleted cells (Fig. 4c), emphasising that the syntenin-1 dependent pathway is crucial for the infection cycle of various HPV types. As with CD63, control experiments showed that trafficking of transferrin and EGF was not affected in cells depleted of syntenin-1 (Supplementary Figs S2 and S3).

**Figure 5:**
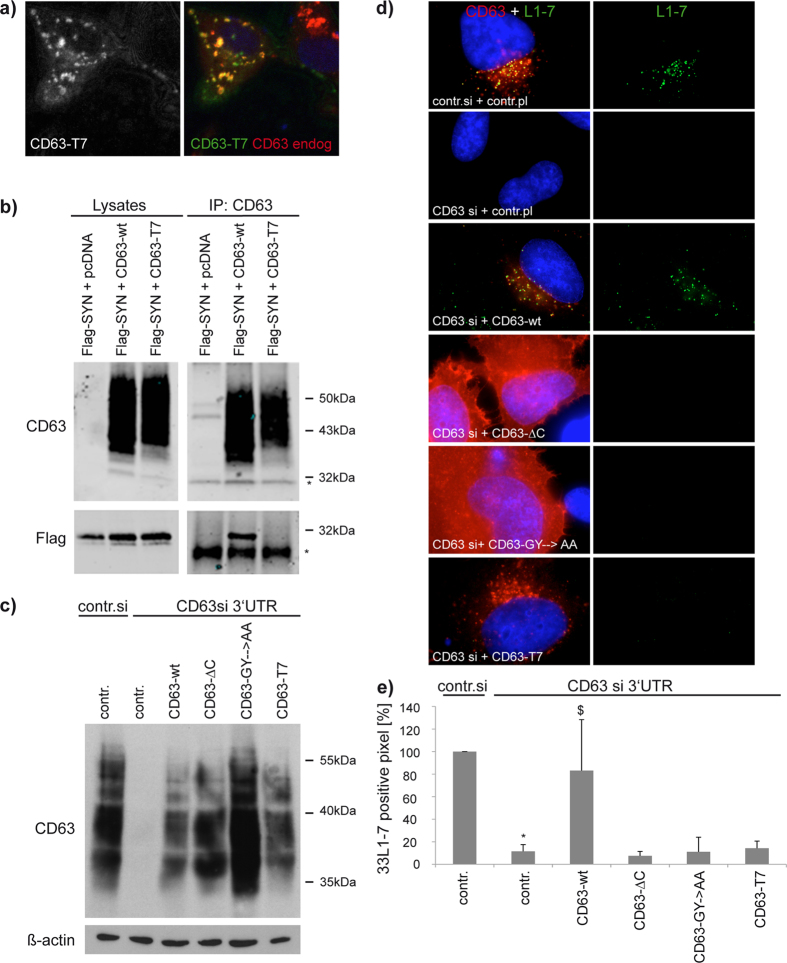
Syntenin-1-binding deficient mutants of CD63 fail to recover L1-7 reactivity. (**a**) Representative pictures of wild-type (wt) and mutant CD63. Rat1 cells were transfected with a plasmid encoding CD63 mutant T7 (human) and stained with species-specific CD63 antibodies, sc-5275 (human) and AD1 (rat). CD63-T7 mutant (green) colocalises with endogenous rat CD63 (red). (**b**) CD63-T7 mutant cannot interact with syntenin-1. CHO cells were cotransfected with plasmids encoding flag-tagged syntenin-1 and human CD63 as indicated. Immunoprecipitation was carried out using mouse anti-human CD63 mAb 6H1. Precipitated proteins were detected by Western blotting using rabbit Flag (lower panels) or CD63 antibodies (upper panels). Lysates were loaded as positive controls for transfection. Asterisks mark non-specific bands. (**c**–**e**) Recovery of L1-7 reactivity using CD63 mutants. CD63 depleted HeLa cells were transfected with control or mutant CD63 plasmid and infected with HPV16. (**c**) CD63 mutants efficiently reexpress CD63 after respective knockdown. (**d**) Shows representative pictures of CD63 and L1-7 double staining in different CD63 mutants. (**e**) L1-7 reactivity was quantified as in Fig. 2e,f. *P < 0.05 significant decrease compared to control, ^$^*P* < 0.05 significant increase compared to cells transfected with CD63 siRNA and control plasmid.

To examine whether CD63 endosomal localisation or formation of the CD63-syntenin-1 complex is important in HPV infection, L1-7 reactivity assays were performed by immunofluorescence after knockdown of endogenous CD63 and subsequent DNA transfection with CD63 wild type or CD63-ΔC, CD63-GY→AA and CD63-T7 mutants. We previously reported that the deletion of the last two C-terminal cytoplasmic amino acids of CD63 (CD63-ΔC mutant) abolished syntenin-1 interaction and CD63 internalization^50^. The CD63 GY→AA mutant in which critical glycine and tyrosine residues in the Tyr-based sorting motif were substituted for alanines also predominantly localise at the plasma membrane^50^. Therefore, we examined the activity of the CD63-T7 mutant, in which the positions of the last two amino acids of CD63 (i.e. V-M was changed to M-V) were interchanged. We found that while colocalising with the endogenous CD63 (Fig. 5a), this mutant did not interact with syntenin-1 (Fig. 5b). As illustrated in Fig. 5d,e, neither CD63-ΔC nor CD63-GY→AA nor CD63-T7 mutants could recover L1-7 reactivity in CD63-depleted HeLa cells. By contrast, staining with the polyclonal L1-antibody K75 was unaffected (Supplementary Fig. S5). Syntenin-1 depletion led to comparable reduction of PsV infection (Fig. 4a) and L1-7 reactivity (Fig. 5e). Together, these experiments reveal that CD63 endosomal localisation and the association with syntenin-1 is critical for CD63-dependent steps needed for HPV16 infectivity.

**Figure 6:**
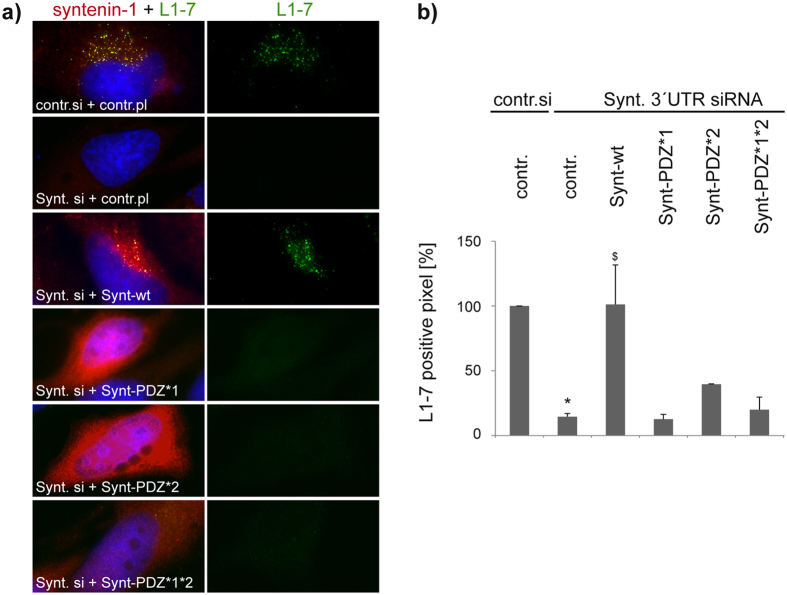
CD63-binding deficient mutants of syntenin-1 fail to recover L1-7 reactivity. Syntenin-1-depleted HeLa cells were transfected with control or syntenin-1 expression plasmid as indicated, infected with HPV16 and sequentially stained with 33L1-7 and syntenin-1 antibodies. L1-7 reactivity assays were performed as in Fig. 2e,f. (**a**) Shows representative pictures of 33L1-7 and syntenin-1 immunostaining. (**b**) Shows quantification of L1-7 reactivity. *P < 0.05 significant decrease compared to control, ^$^*P* < 0.05 significant increase compared to cells transfected with CD63 siRNA and control plasmid.

To further emphasise the significance of CD63-syntenin-1 interaction in HPV infection, we performed recovery assays of virus L1-7 reactivity using syntenin-1 mutants (Supplementary Fig. S6). Syntenin-1 contains two PDZ domains which both contribute to CD63 interaction and a critical point mutation in either PDZ1 (Gly^126^→Asp) or PDZ2 (Gly^210^→Glu) compromises the assembly of the CD63-syntenin-1 complex^50^. Syntenin-1 PDZ mutants (Synt-PDZ*1, -PDZ*2, and -PDZ*1*2) were transiently expressed in syntenin-1-depleted cells (Supplementary Fig. S6) and post-uncoating processing of the viral capsid was monitored as above (Fig. 6a,b). None of these syntenin-1 mutants could recover L1-7 reactivity. In other experiments we found that overexpression of syntenin-1 PDZ mutants in the control cells had no significant effect on L1-7 reactivity (Supplementary Fig. S6). Importantly, robust staining of virions with K75 Ab indicated that the pre-disassembly steps of infection were unaffected by syntenin-1 depletion (Supplementary Fig. S7). Again, these results indicate that CD63-syntenin-1 complex formation is required for HPV infection.

### CD63-syntenin-1-complex controls viral trafficking to multivesicular endosomes

**Figure 7:**
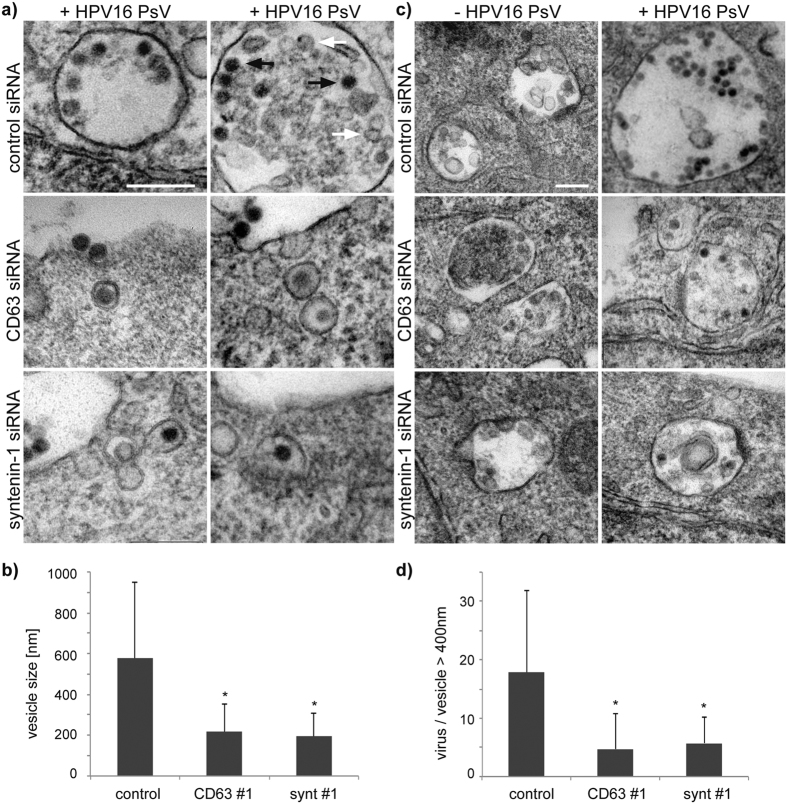
Ultrastructural analyses of HPV16-filled endosomes. (**a–d**) HeLa cells were treated with CD63 or control siRNA and infected with HPV16 PsV. (**a**) Representative electron micrographs illustrating the morphology of virus containing endosomes (black arrow: HPV16 PsV; white arrow: intraluminal vesicles). Scale bar represents 200 nm. **(b)** For quantification of the size (diameter) of virus containing endosomes, 20 micrographs of target and control siRNA-treated cells were measured. **(c)** Representative electron micrographs of virus containing endosomes (diameter > 400 nm) after mock or HPV16 PsV infection. Scale bar represents 200 nm. **(d)** Quantification of viruses per vesicle with diameter >400 nm was determined by analysing 20 micrographs of control or target siRNA-treated cells, respectively. *P < 0.05 compared to control.

**Figure 8:**
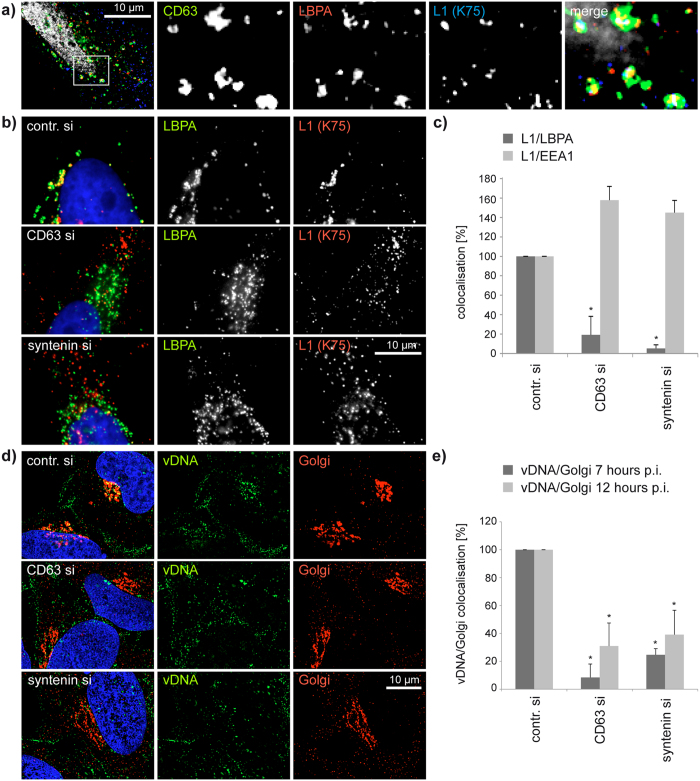
Trafficking of HPV16 to multivesicular endosomes requires CD63/syntenin-1 complex. (**a**) Representative pictures of CD63-GFP (green), LBPA (red), and HPV16 L1 (blue) triple staining with LBPA mAb and L1 pAb K75 antibodies. (**b,c**) Colocalisation analysis of HPV16 L1 and LBPA. HeLa cells were transfected with control siRNA, CD63 siRNA or syntenin-1 siRNA and infected with HPV16 PsV and stained with LBPA mAb and L1 pAb K75 antibodies. (**b**) Shows representative pictures of L1 (red) and LBPA (green) colocalisation. Nuclei are shown in blue. (**c**) Colocalisation of LBPA and L1 as well as early endosome marker EEA1 and L1 was analysed as in Fig. 3e (*P* = 0,062 for CD63 siRNA and 0,069 for syntenin-1 siRNA). (**d,e**) Colocalisation analysis of viral DNA and the Golgi. HeLa cells were transfected with control, CD63 or syntenin-1 siRNA and infected with EdU-HPV16 PsV for 7 or 12 hours. (**d**) Shows representative pictures of viral DNA (vDNA, green) and Golgi (red) 12 hours post infection. (**e**) Colocalisation of viral DNA and Golgi was analysed as above and normalized to infected control siRNA treated cells. *P < 0.05 compared to control.

To examine the role of the CD63-syntenin-1 complex in intracellular trafficking in more detail, we compared distribution of internalised HPV16 PsV in control and CD63- or syntenin-1-depleted cells using electron microscopy. These experiments revealed that in control cells 7 hours post-initiation of HPV infection a significant proportion of viral particles was found in multivesicular endosomes whose diameter exceeded 400 nm (Fig. 7a). By contrast, in CD63- or syntenin-1-depleted cells the viral capsids were located in smaller endocytic vesicles (i.e. <250 nm in diameter) in the cell periphery (Fig. 7b). Consistently, endosomes larger than 400 nm in diameter contained lower numbers of viral particles in CD63- or syntenin-1-depleted cells as compared to control cells (Fig. 7c), underscoring the involvement of the CD63-syntenin-1 complex in trafficking of internalised HPV to MVEs. The overall number and size of MVEs were not affected in CD63-, or syntenin-1-depleted cells and independent of PsV infection (Fig. 7c). In agreement with the role of CD63-syntenin-1 complex in endocytic trafficking of HPV we observed that colocalisation between L1 and LBPA, a well-established marker of MVEs^51^, was markedly decreased in both CD63- and syntenin-1-depleted cells when compared to control cells (Fig. 8a–c). Furthermore, in CD63- and syntenin-1-depleted cells, PsV were distributed more towards the cell periphery and colocalisation with early endosomal marker EEA1 was increased (Fig. 8bc), providing an additional confirmation that HPV16 trafficking to perinuclear MVEs depends on the CD63-syntenin-1-complex. Moreover, transport of viral DNA to the Golgi apparatus was significantly decreased in CD63 and synenin-1-depleted cells, indicating that CD63-syntenin-1 mediated HPV trafficking steps to MVEs precede the retrograde trafficking of viral DNA to Golgi (Fig. 8d,e).

### Syntenin-1 regulates HPV infection via ALIX interaction

**Figure 9:**
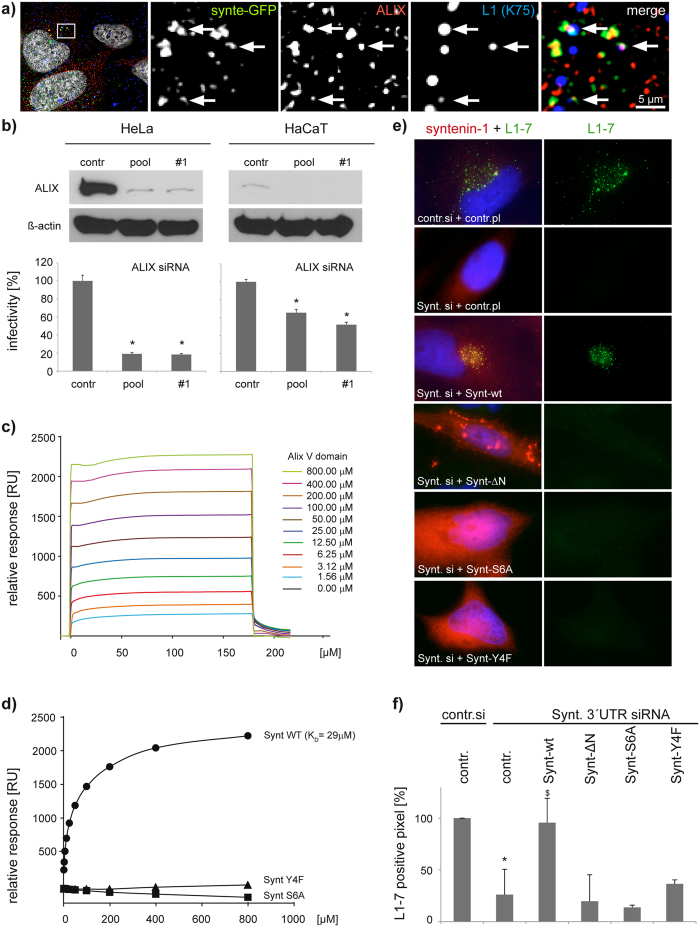
ALIX interacts with syntenin-1 and is required for papillomavirus infection. (**a**) HPV16 L1 colocalises with syntenin-1 interacting protein ALIX. Representative pictures of ALIX-HA and syntenin-GFP overexpression show colocalisation of syntenin-1 (green), ALIX (red) and L1 (blue) marked by arrows. (**b**) ALIX knockdown correlates with reduced infectivity. Upper panels show efficiency of ALIX knockdown in HeLa and HaCaT cells. Bottom panels show HPV16 infection assay of HeLa and HaCaT cells after ALIX siRNA treatment. Infectivity was measured as in Fig. 2b. (**c,d**) Affinity of ALIX V-domain-syntenin1 interaction by SPR equilibrium binding analysis. (**c**) Double referenced sensorgrams are shown as a plot of relative response of ALIX V-domain/syntenin-1 N-terminal wild-type peptide (654 RU) immobilized surfaces over time. Streptavidin coated non-peptide immobilized surface served as reference. RU, Response units. (**d**) Data points of ALIX V-domain binding to syntenin-1 wild-type peptide (filled circle), Y4F peptide (filled triangle) or S6A peptide (filled square) fitted to a one-site binding model. Syntenin-1 wild-type peptide showed a KD of 29 μM. (**e,f**) Syntenin-1-depleted HeLa cells were transfected with control, syntenin-1 wild-type or syntenin-1 mutant expression plasmid. L1-7 reactivity assay was performed as in Fig. 2e,f. (**e**) Shows representative pictures. (**f**) Shows quantification of L1-7 reactivity. *P < 0.05 compared to control, ^$^*P* < 0.05 compared to cells transfected with CD63 siRNA and control plasmid.

We investigated whether CD63-syntenin-1-dependent HPV trafficking requires recruitment of additional proteins to enable successful HPV infection. Previous studies demonstrated that ALIX also interacts with components of the ESCRT machinery and that these interactions may be critical for the biogenesis of MVEs^44^,^52^. As ALIX is also a syntenin-1 interacting protein, we hypothesised that ALIX is recruited to the CD63-syntenin-1 complex during the transition of viral particles to MVEs. Indeed, when analysed by immunofluorescent stainings, ALIX showed a prominent colocalisation with syntenin-GFP and HPV16 L1 in vesicular structures (Fig. 9a). These data suggest that ALIX may function in the context of the CD63-syntenin-1 complex to ensure viral trafficking through MVEs. Accordingly, we found that depletion of ALIX significantly reduced HPV infectivity rate (Fig. 9b, control in Supplementary Fig. S1). The N-terminal ^3^LYPSL^7^ sequence of syntenin-1 was shown to be critical for its interaction with ALIX^44^, a finding which we could reproduce using surface plasmon resonance (SPR) experiments. Here, ALIX shows strong binding to the peptide corresponding to the first fourteen amino acids of syntenin-1 including the LYPSL sequence (Fig. 9c). This binding was impeded when we used syntenin-1-derived peptides with point mutations at tyrosine 4 (Y4F) or serine 6 (S6A) of the LYPSL sequence (Fig. 9d). Accordingly, Synt-ΔN (a mutant lacking the first 100 AA), Synt-Y4F, and Synt-S6A mutants were unable to restore early steps of viral infection when reexpressed in syntenin-1 depleted cells (Fig. 9e,f, Supplementary Fig. S6 and controls in Supplementary Fig. S7). Interestingly, we found that overexpression of Synt-ΔN and Synt-S6A has a suppressive effect on L1-7 reactivity when overexpressed in control cells (Supplementary Fig. S6) thus further supporting the importance of the ALIX-binding site in HPV trafficking. These results demonstrate that the CD63-syntenin-1 complex requires ALIX to control the post-entry pathway in HPV infection.

## Discussion

Previous data revealed that various oncogenic human papillomaviruses (HPV) enter cells via a novel endocytosis mechanism^22^,^24^,^26^,^40^. Here, the post-endocytic intracellular pathway controlling virus capsid disassembly and infection was investigated. We identified the CD63-syntenin-1-ALIX complex as a novel factor in the viral replication cycle regulating post-endocytosis trafficking of viruses to multivesicular endosomes. Having previously established the contribution of tetraspanin-enriched microdomains in HPV cell entry, our current data emphasise the critical role of tetraspanin-based protein platforms in regulation of various aspects of HPV infection.

We have recently reported that the association with other tetraspanins is essential in the CD151-dependent HPV infection pathway^23^. As CD151 controls viral uptake, other tetraspanins may either facilitate this process, or function downstream by directing trafficking of internalised viruses along the endocytic pathway. Here, we present evidence that the latter is regulated by a complex of tetraspanin CD63 and its interaction partner syntenin-1. These results provide a novel mechanistic insight into the poorly characterised process of post-endocytosis HPV trafficking. We demonstrated that HPV16 is physically linked to a CD63-containing protein complex and that the delivery of endocytosed viral particles to multivesicular endosomes was repressed in cells depleted of CD63 or syntenin-1 while MVE biogenesis was not affected. These observations are in line with an earlier report describing that lack of CD63 does not lead to any marked morphological alterations of the late endosomal/lysosomal compartment^53^. Furthermore, depletion of these trafficking components led to decreased transport of the viral DNA to the Golgi implying that the CD63-syntenin-1-complex controls the transition of virus-containing endosomes to acidic cellular compartments, a critical step preceding retrograde trafficking of HPV to the Golgi.

With its physical proximity to HPV receptors within tetraspanin-enriched microdomains (e.g. EGFR, annexin A2, laminin-binding integrins^6^,^41^,^54^,^55^,^56^) and interaction capacity with syntenin-1 at virus containing endosomes, CD63 provides a critical physical link between internalised viral particles and intracellular endocytic machinery represented in the complex by syntenin-1 (see below). CD63 has been previously implicated in endocytic trafficking of various transmembrane proteins (e.g. CXCR4, VEGFR2, synaptotagmin VII) via mechanisms involving its tyrosine-based sorting signal (G-Y-E-V-M sequence)^57^,^58^,^59^. As expected^50^, we found that mutation of this sequence (in CD63-GY→AA mutant) stabilised CD63 on the plasma membrane and precludes recruitment of the protein to HPV-containing endosomes. Importantly, the CD63-GY→AA mutant was unable to support HPV infection thereby suggesting that the CD63-dependent step in virus infection relies on the endosomal CD63 protein pool.

Progressive recruitment of syntenin-1 to virus-containing endosomes and retention of internalised HPV in small peripheral endosomes in syntenin-depleted cells supports the idea that the CD63-syntenin-1 complex controls the pathway directing internalised viruses towards multivesicular endosomes. Accordingly, we observed that CD63 and syntenin-1 knockdown lead to reduced degradation of viral capsid proteins (data not shown). The role of syntenin-1 in various aspects of endocytic trafficking is well established^42^,^43^,^60^,^61^. Its ability to link numerous transmembrane partners to key components of the endocytic molecular machinery (i.e. phospholipids, small GTPases, ubiquitylated proteins) is likely to be central in the diverse endocytic functions of syntenin-1. Our results demonstrate that syntenin-1 partner protein ALIX is required for productive HPV infection. Interestingly, interaction between syntenin-1 and ALIX is critical for the production of exosomes, nanovesicles released by cells after MVEs fuse with the plasma membrane^44^. This, and other overlapping functions of CD63, syntenin-1 and ALIX such as viral egress from cells, ubiquitin-independent sorting of transmembrane cargos to MVEs^52^,^62^, suggest diverse cellular functionality of the complex. Previous studies demonstrated that ALIX interacts with TSG101^63^,^64^, an ESCRT-I protein binding to the HPV minor capsid component L2^32^. Therefore, spatial juxtaposition of ALIX with endocytosed viruses may be important for the subsequent egress of disassembled virions from MVEs to the Golgi.

We propose that syntenin-1 has a central role in regulating the functionality of the CD63-syntenin-ALIX complex in HPV infection. Indeed, recent reports demonstrated that syntenin-1 can be transiently phosphorylated at Tyr4 by the Src kinase^65^ and downstream of activated FGFR2^66^, both of which were previously linked to HPV infection^18^,^21^,^26^,^67^. Our data indicate that this modification is likely to control the dynamics of syntenin-1-ALIX binding preventing interaction during the earlier steps of viral cell entry. Likewise, the assembly of the syntenin-1-ALIX complex may be suppressed by ULK1-induced phosphorylation of syntenin-1^68^. ULK1 is a serine-threonine kinase which was shown to play a critical role in the initiation of autophagy^69^,^70^. Autophagy pathway activation and subsequent dissociation of the CD63-syntenin-ALIX complex may divert trafficking of internalised viral particles from MVEs to other endolysosomal compartments formed under environmental insults. The proposed regulatory role of syntenin-1 phosphorylation is in agreement with Schelhaas and colleagues finding that broad range inhibitors of tyrosine and serine/threonine phosphatases suppressed HPV entry^26^. Finally, syntenin-1 was shown to interact with Rab5 and Rab7^71^, key regulators of endocytic trafficking, which are suggested to control endocytic routes of internalised HPV^26^,^33^.

**Figure 10:**
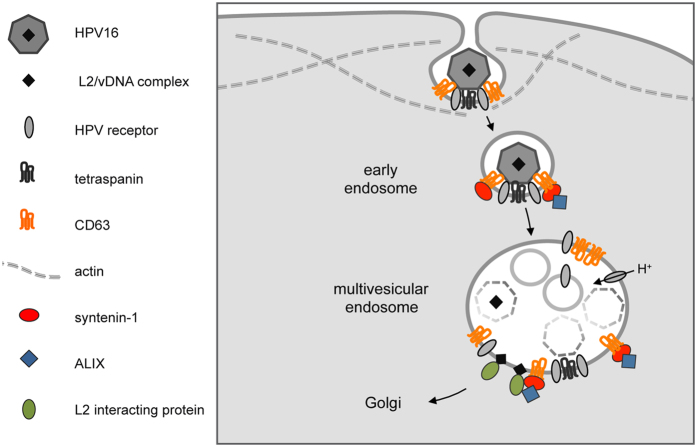
Model of CD63-syntenin-ALIX-dependent trafficking pathway of internalized HPV16. HPV16 is endocytosed via tetraspanin-enriched microdomains. Virus uptake results in recruitment of syntenin-1 and ALIX. CD63-syntenin-ALIX complex formation regulates HPV post-endocytic trafficking to multivesicular endosomes, which is a precondition for HPV capsid disassembly, transport of the L2/vDNA complex to the Golgi compartment, and infection.

In conclusion, our study presents the CD63-syntenin-ALIX complex as a principal regulator of post-endocytic trafficking of HPV to multivesicular endosomes (Fig. 10). These compartments contain factors such as the V-ATPase, required for virus disassembly^30^,^31^, and cyclophillins, required for separation of L1 and L2^29^, which enables interaction of L2 with cytosolic proteins to facilitate retrograde transport to the nucleus^30^,^34^,^35^,^36^,^39^,^72^,^73^. Within the CD63-syntenin-1-ALIX-complex, syntenin-1 may represent the key factor integrating intracellular signalling pathways and ensuring their spatio-temporal coordination during early steps of HPV infection. Further analysis of the molecular network involving syntenin-1 is needed to draw a comprehensive map of trafficking routes directing endocytosed HPV to MVEs for productive infection.

## Materials and Methods

### Cell lines and Pseudovirions

Normal human epidermal keratinocytes (NHEK) were purchased from PromoCell, Heidelberg, Germany and cultivated according to manufacturer’s instructions. The human cervical carcinoma cell line HeLa was purchased from the German Resource Centre for Biological Material (DSMZ), Braunschweig, Germany. HaCaT cells (human non-virally immortalised keratinocytes) were obtained from Cell Lines Services (CLS), Eppelheim, Germany. The cells were grown at 37 °C in Dulbecco’s modified Eagle’s medium (DMEM) supplemented with 10% foetal calf serum (FCS), 1% Glutamax I (Invitrogen, Carlsbad, CA, USA), 1% modified Eagle’s medium nonessential amino acids and antibiotics. CHO-K1 cells were grown in Ham’s F-12 media supplemented with 10% fetal calf serum.

HPV16, -18, and -31 pseudovirions (PsV) were prepared as previously described^74^. Briefly, expression plasmids carrying codon-optimized L1 and L2 expression vectors were cotransfected with a pcDNA3.1/Luciferase reporter plasmid into 293TT cells^39^,^75^. L1 and L2 expression vectors pUF3/hu16L1, pUF3/hu16L2, pe18L1bhb, pe18L2bhb, p31L1h and p31L2h for HPV16, -18 and -31 were kindly provided by Martin Müller, Heidelberg, Germany, Chris Buck, Bethesda, MD, and Tadahito Kanda, Tokyo, Japan, respectively^24^,^76^,^77^,^78^. 48 hours post-transfection, the cells were processed by lysis and nuclease digestion. The pseudovirions were purified from the cell lysates by OptiPrep (Sigma-Aldrich, St. Louis, MO, USA) gradient centrifugation. Quantification of pcDNA3.1/Luciferase positive pseudovirions was performed as described^23^,^39^. Optional labeling of the pseudogenomes with 5-ethinyl-2′-deoxyuridine (EdU) was performed by supplementing the growth medium with 100 μM EdU 6 h post transfection^79^.

### Statistics

All experiments were reproduced at least three times. All analysable data points were included in statistical analysis. Statistical differences between groups and statistical graphs were assessed with a two-tailed, paired t-test using Microsoft Office Excel 2016. Differences between groups were considered significant when the P-value was <0.05.

### Plasmids and Antibodies

Expression plasmids encoding CD63 and syntenin-1 were described previously^50^,^80^,^81^. CD63/T7 was generated using a standard PCR protocol generating a CD63 protein which resembles the C-terminus of Tspan7 (primers are available upon request). CD63 was amplified from clone IRAUp969A121D6 (imaGenes, Berlin, Germany) by standard PCR. The CD63 sequence was then cloned into the XhoI-KpnI site of the pEGFP-C1 (Clontech, Heidelberg, Germany) vector. The ALIX-HA containing plasmid (pCAGGS-HA-AIP1) was a kind gift from Prof. Takemasa Sakaguchi (Hiroshima, Japan)^82^. The ALIX V-domain containing plasmid (pGST2-ALIX^364–716^) was a kind gift from Prof James Hurley (NIH, USA). The HPV16 L1-specific antibodies mouse mAbs L1-7, H56.E, and 16L1-312F and rabbit pAb K75 (detecting HPV16, 18, and 31) have been described previously^22^,^24^,^29^,^47^,^83^. The CD63 antibody 6H1 was described previously^84^, the CD63 mAb sc-5275, rat CD63 mAb AD1, syntenin-1 mAb sc-100336, Rab5 mAb sc-46692, mouse Golgin-97 mAb A-21270, ALIX mAb sc-53540, rat HA Ab 3F10 and the mouse mAb anti β-Actin (AC-15) were purchased from Santa Cruz (Dellas, TX, USA), Molecular Probes (Eugene, OR, USA), Roche (Basel, Switzerland), and Sigma-Aldrich (St. Louis, MO, USA), respectively. The LBPA mAb was kindly provided by Jean Gruenberg, Geneva, Switzerland.

### Immunofluorescence microscopy

NHEK or HeLa cells were grown on coverslips. After transfection and/or infection (with approximately 200 particles per cell), cells were treated with paraformaldehyde/Triton X-100 or methanol. For LBPA staining, cells were fixed with 4% paraformaldehyde, and afterwards incubated with 50mM NH_4_Cl. Fixed cells were stained with the indicated primary antibodies (LBPA in the presence of 0,5% saponin^51^) and with Alexa-conjugated species-specific secondary antibodies (Invitrogen, Carlsbad, CA, USA). DNA was stained with Hoechst 33342 (Sigma-Aldrich, St. Louis, MO, USA). Coverslips were mounted onto slides using Fluoprep mounting medium (bioMérieux). Images were acquired using the LSM 710 (Carl Zeiss, Jena, Germany) and the LSM software ZEN 2008 or using a Zeiss Axiovert 200 M microscope equipped with a Plan-Apochromat 100x (1.4 NA) and Axiovision deconvolution and colocalisation software 4.7 (Carl Zeiss, Jena, Germany). Image files were assembled into figures using InDesign software (Adobe). For colocalisation analysis, cells were grown on coverslips and/or transfected with siRNA. Cells were infected with pseudoviruses for 7 or 8 (EdU-viruses) hours and stained with antibodies as indicated. Colocalising pixels of at least 10 images (3–5 cells per image) were analysed using Colocalisation Software 4.7 (Carl Zeiss, Jena, Germany).

### Knockdown of CD63, syntenin-1, and ALIX

The CD63-specific siRNAs CD63#1 (GUUCUUGCUCUACGUCCUCdTdT, Sigma-Aldrich, St. Louis, MO, USA), CD63 3′UTR (GGUUUUUCAAUUAAACGGAdTdT, Sigma-Aldrich, St. Louis, MO, USA), CD63#2 (GGAGAACUAUUGUCUUAUGdTdT, Sigma-Aldrich, St. Louis, MO, USA), syntenin-1 #1 (HS_SDCBP_2, Qiagen, Hilden, Germany), syntenin-1 3′UTR (HS_SDCBP_4, Qiagen, Hilden, Germany), syntenin-1 #3 (HS_SDCBP_5, Qiagen, Hilden, Germany), syntenin-1 #4 (smart pool of four siRNAs: GGAGAGAAGAUUACCAUGA, GACCAAGUACUUCAGAUCA, GGAUGGUCUUAGAAUAUUU, GCAUUUGACUCUUAAGAUU, Dharmacon, Lafayette, CO, USA). The ALIX-specific siRNAs pool (smart pool, M-004233-02, Dharmacon, Lafayette, CO, USA), ALIX #1 (GCAGTAATATGTCTGCTCA^85^). Nonsilencing control siRNA (AllStars Neg. Control siRNA) was obtained from Qiagen, Hilden, Germany. The 3′UTR siRNAs are targeting the 3′UTR of the target genes and are not able to silence CD63 or syntenin-1 plasmid expression. HeLa, HaCaT or NHEK cells were transfected with 30 nM of siRNA using RNAiMAX (Invitrogen, Carlsbad, CA, USA) according to manufacturer’s instructions. Subsequent experiments were done 24 hours or 48 hours after siRNA transfection.

### Pseudovirus infection assay

Cells were grown in 24-well plates and treated with CD63-, syntenin-1 or ALIX siRNA for 48 hours. Cells were infected with 100 (HeLa and HaCaT) or 500 (NHEK) luciferase vector-positive pseudovirions per cell^39^,^75^. Infection efficiencies of HPV pseudovirions were assessed by quantification of luciferase expression 24 (HeLa and HaCaT) or 48 hours (NHEK) post infection. Luciferase expression control and pseudovirus infection assay were performed as described in Scheffer *et al*.^23^.

### Recovery experiments

HeLa cells were treated with CD63- or syntenin-1-specific siRNA targeting the 3′UTR of the gene or control siRNA for 24 hours and transfected with the indicated plasmids encoding CD63 or syntenin-1-wt and various mutants or control plasmid (pZeoSV or pCMV-HA) using Lipofectamine 2000 (Invitrogen, Carlsbad, CA, USA) for additional 24 hours. Afterwards, the cells were infected with HPV16 pseudovirions for 7 hours and infection or L1-7 reactivity assay was performed.

### Coimmunoprecipitation assay

HeLa cells were seeded on 100-mm dishes in duplicate and cultured overnight. Cells of one dish were infected with HPV16 PsVs for five hours. Dishes were put on ice, cells were washed with Hepes buffer (25 mM Hepes, 150 mM NaCla, 5 mM MgCl_2_) and lysed in 1% Chaps/Hepes buffer with protease inhibitors Aprotinin and Leupeptin (10 μg/ml each). Lysates were incubated for 15 minutes at 4 °C on a rotating wheel, treated with an ultrasonifier three times for 20 seconds each (30% duty cycle; output control, 30%; Branson Sonifier 250, Emerson Industrial Automation, St. Louis, MO, USA) and incubated for 30 minutes at 4 °C on a rotating wheel. Dynabeads (M280 Sheep anti-Rabbit IgG, Invitrogen, Carlsbad, CA, USA) were prepared according to manufacturer’s instructions and incubated with K75 rabbit IgG for 20 minutes at room temperature on a rotating wheel. Cell lysates were incubated with K75-coupled Dynabeads overnight at 4 °C on a rotating wheel. Beads were washed in lysis and Hepes buffer. Likewise, CHO cells were seeded and transfected with Flag-tagged syntenin-1 and/or human CD63 plasmids. At 48 hours post-transfection, cells were lysated by immunoprecipitation buffer (0.5% Brij 98–0.5% Triton X-100– PBS, 2 mM phenylmethylsulfonyl fluoride, 10 μg/ml Aprotinin and Leupeptin) for 4 to 16 hours at 4 °C. Undissolved material was pelleted at 12,000 rpm for 10 min. Immune complexes were collected by incubation of cellular lysate with anti-CD63 mAb (6H1) pre-bound to protein G agarose beads for 16 hours and washed four times with immunoprecipitation buffer. Hela and CHO cell precipitates were boiled in Laemmli sample buffer and processed for Western blotting with anti-bodies as indicated in figure legends. CD63 Western blots were performed under non-reducing conditions.

### Cell binding assay by Western blotting

HeLa cells grown in 12-well plates were transfected with siRNAs for 48 hours. Subsequently, cells were incubated with HPV16 PsV for 1 hour at 37 °C, washed four times with PBS and then collected in SDS sample buffer for Western blotting. Cell-bound HPV16 particles were stained with anti-L1 antibody 312F and β-Actin was stained as input control.

### Detection of surface bound particles by flow cytometry

HeLa cells were transfected with the indicated siRNAs and/or plasmids and infected with HPV16 PsV for 1 hour (cell binding assay). Subsequently, cells were extensively washed with PBS to remove unbound virions and analysed by flow cytometry or incubated for additional 23 hours. Cells were trypsinized with 0,25% Trypsin/2,5mM EDTA. Surface-bound particles were stained using the anti-L1 (K75) antibody and secondary anti-rabbit Alexa Fluor-488 antibody. Background staining was determined using a non-specific IgG serum. Measurements of surface bound particles at 1 hour post virus addition served as virus-cell binding analysis (see above) and comparison of bound particles at 1 h versus 24 h to show virus disappearance from the cell surface by endocytosis.

### Detection of L1-7 epitope by immunofluorescence

HeLa cells were grown on coverslips and transfected with siRNAs. 48 hours later cells were infected with HPV16 pseudovirions (with approximately 200 particles per cell) and incubated for 7 hours at 37 °C. Subsequently, cells were fixed with methanol and processed for staining with mAb 1-7 as described previously^22^. This mAb recognizes a specific epitope located in the interior of the pseudovirion capsid and is not accessible in intact virions. The samples were analysed by fluorescence microscopy using a Zeiss Axiovert 200M inverted microscope (Carl Zeiss, Jena, Germany) and quantified by ImageJ software. For quantification, the relative amount of internalised particles was determined based on the L1-7 positive pixels relative to the cell nucleus signal (DNA/Hoechst 33342 positive pixels) out of 100 randomly selected cells (knockdown experiments) and 100 CD63 or syntenin-1 expressing cells (recovery experiments) from at least four independent experiments. A threshold value was set to exclude background.

### Preparation of endosomes

4.5 × 106 HeLa cells were either left untreated or were infected with 50 vge/cell of HPV16 PsV for 4 or 7 hours. Endosomal fractions were prepared as described^86^,^87^. Briefly, cells were harvested and homogenized, a post-nuclear supernatant was prepared and adjusted to 40.6% sucrose, 3 mM imidazole, pH 7.4, loaded at the bottom of an SW60 tube, and overlaid sequentially with 35% and 25% sucrose solutions in 3 mM imidazole, pH 7.4, and homogenization buffer (HB; 8.5% sucrose, 3 mM imidazole, pH 7.4). The gradient was centrifuged for 90 min at 14 000 × g. Thirteen fractions were collected. Early endosomal fractions (35%/25% interface) were identified by immunoblotting using the endosomal marker Rab5^87^,^88^.

### Quantitative mass spectrometry

For protein digest early endosomes were pelleted by ultracentrifugation (100.000 × g, 1 h, 4 °C), proteins reduced by adding 5 mM DTT, free cysteines alkylated with iodoacetamide (Sigma-Aldrich, St. Louis, MO, USA), and proteins digested with 0.2 μg porcine sequencing grade trypsin (Promega, Mannheim, Germany)^89^. Nanoscale liquid chromatography of tryptic peptides was performed with a Waters NanoAcquity UPLC system equipped with a 75 μm × 150 mm BEH C18 reversed phase column and a 2.6 μl PEEKSIL-sample loop (SGE, Darmstadt, Germany). Mass spectrometry analysis of tryptic peptides was performed using a Waters Q-TOF Premier API system, operated in V-mode with typical resolving power of at least 10,000. All analyses were performed using positive mode ESI using a NanoLockSpray source. For data processing and protein identification the continuum LCMSE data were processed and searched using the IDENTITYE- Algorithm of ProteinLynx Global Server (PLGS) version 2.3. The resulting peptide and protein identifications were evaluated by the software using statistical models similar to those described by Skilling *et al*.^90^. Protein identifications were assigned by searching the UniProtKB/Swiss-Prot Protein Knowledgebase Release 52.3.

### Electron Microscopy

HeLa cells were infected with HPV16 PsV (approx. 500 p/cell) and after 7 hours fixed with 2.5% glutaraldehyde in PBS for 45 minutes at room temperature. Cells were washed with PBS, harvested and embedded in Epon 812 according to standard procedures. 70 nm ultrathin sections were cut, stained with 1% lead citrate and 2% uranyl acetate and finally analysed in a Zeiss CEM 902 electron microscope (Carl Zeiss, Jena, Germany), equipped with TRS digital camera. For quantification, 20 pictures of each sample were analysed.

### Surface Plasmon Resonance

The interaction of ALIX and syntenin-1 was quantified using a BIAcore 3000 instrument (GE Healthcare) in 10 mM HEPES (pH 7.4) buffer, 150 mM NaCl and 0.005% Surfactant P20. Streptavidin (Sigma-Aldrich, St. Louis, MO, USA) was covalently coupled to CM5 sensor chips (GE Healthcare) via primary amines using the standard amine coupling kit (GE Healthcare). For coupling, streptavidin was injected at 0.5 mg·mL^−1^ in 10 mM sodium acetate (pH 5.5) with coupling levels ranging from 6000 to 8000 RU. Biotinylated syntenin-1 N-terminal 11-mer peptides (LifeTein LLC, Somerset, NJ, USA) were immobilized at the indicated levels by injection at 20 to 50 mg·mL^−1^ for 0.5 min over streptavidin-coupled surfaces. Equilibrium affinity measurements involved injecting ALIX V-domain (purified as described^91^) over immobilized syntenin N-terminal peptides at 20 μl/min. Data was collected from two replicates and analysed using Scrubber 2 software (Biosensor Tools LLC, Salt Lake City, UT, USA).

## Additional Information

**How to cite this article**: Gräßel, L. *et al*. The CD63-Syntenin-1 Complex Controls Post-Endocytic Trafficking of Oncogenic Human Papillomaviruses. *Sci. Rep.* **6**, 32337; doi: 10.1038/srep32337 (2016).

## Supplementary Material

Supplementary Information
