# Supplementary material for: The CD63-Syntenin-1 Complex Controls Post-Endocytic Trafficking of Oncogenic Human Papillomaviruses
Source: Sci Rep. 2016 Aug 31;6:32337. doi: 10.1038/srep32337 (PMC5006017; doi:10.1038/srep32337)
Supplement: Supplementary Information [file srep32337-s1.pdf]

The CD63-Syntenin-1 Complex Controls Post-Endocytic Trafficking of Oncogenic Human Papillomaviruses

Linda Gräßel[1§], Laura Aline Fast[1§], Konstanze D. Scheffer[1], Fatima Boukhallouk[1], Gilles A. Spoden[1], Stefan Tenzer[2], Klaus Boller[3], Ruzica Bago[4], Sundaresan Rajesh[4], Michael Overduin[5], Fedor Berditchevski[4]*, Luise Florin[1]*

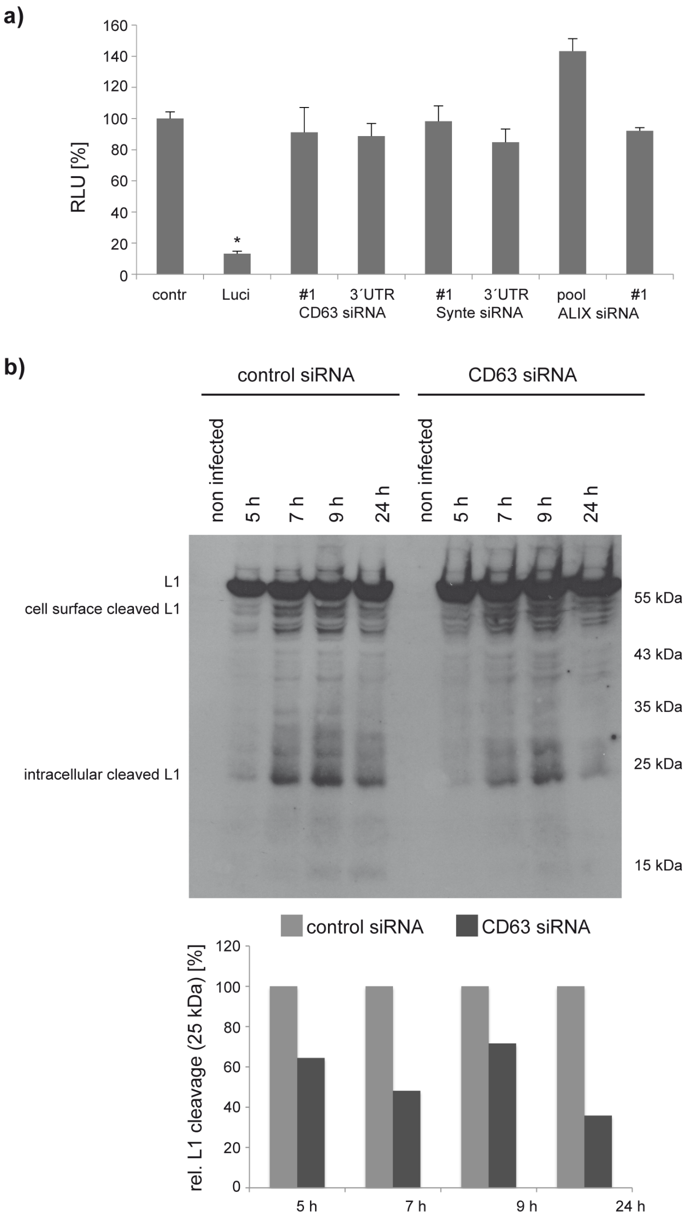

S1 Fig. Influence of CD63, syntenin-1, and ALIX specific siRNAs on luciferase expression and CD63 siRNA on L1-cleavage. (**a**) HeLa cells were co-transfected with control, luciferase-specific, or target-specific siRNA as well as luciferase expressing plasmid as a control for the effect of siRNA transfection on luciferase expression. Luciferase specific siRNA served as a positive control (CTTACGCTGAGTACTTCGAdT, Sigma). (**b,c**) CD63 depletion reduces endosomal L1 cleavage. (**b**) Western blot shows L1 cleavage after 5, 7, 9 or 24 hours HPV16 infection in control or CD63 treated HeLa cells. Endosomal L1 cleavage products <40 kDa are reduced in CD63 depleted cells. (**c**) Shown is the amount of late endosomal L1 cleavage products (~25 kDa) relative to the amount of early cellular cleavage products (~50 kDa) after indicated time points of infection. Relative band intensities were quantified densitometrically.

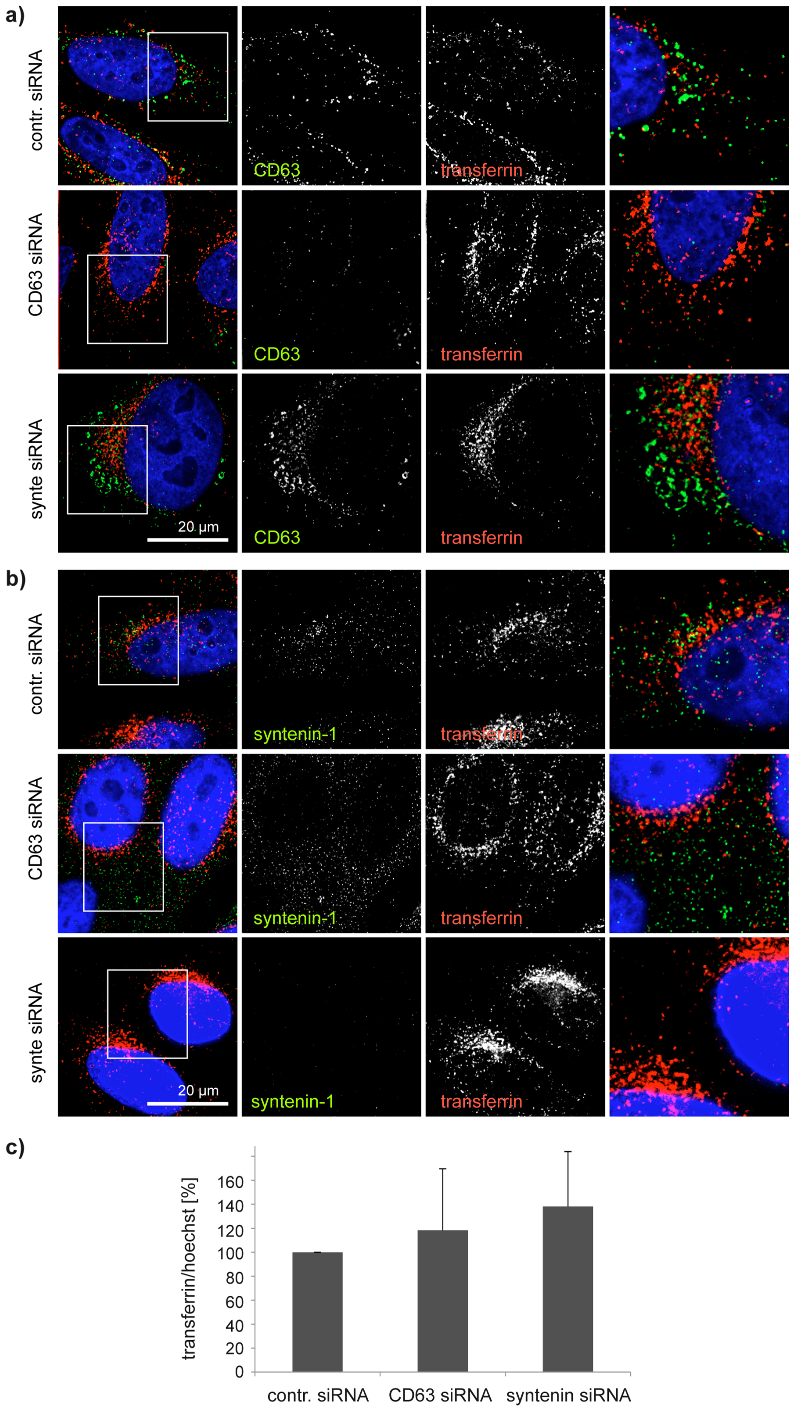

S2 Fig. CD63 and syntenin-1 specific siRNAs have no significant influence on transferrin-uptake and perinuclear accumulation. (**a**,**b**) Hela cells were depleted of CD63 or syntenin-1 or treated with control siRNA. Transferrin-Alexa Fluor 546 (red) was added to cells for 1 hour and analysed by fluorescent microscopy after acid wash and CD63 (green) (**a**) or syntenin-1 (green) immunostaining (**b**). Nuclei are shown in blue. (**c**) Quantification of relative transferrin staining per cell was performed by analysis of transferrin-positive pixels of at least 30 images (3-5 cells per image) using ImageJ-script. Shown are the results of four independent experiments using CD63 and syntenin-1 siRNAs normalized to control siRNA treated cells.

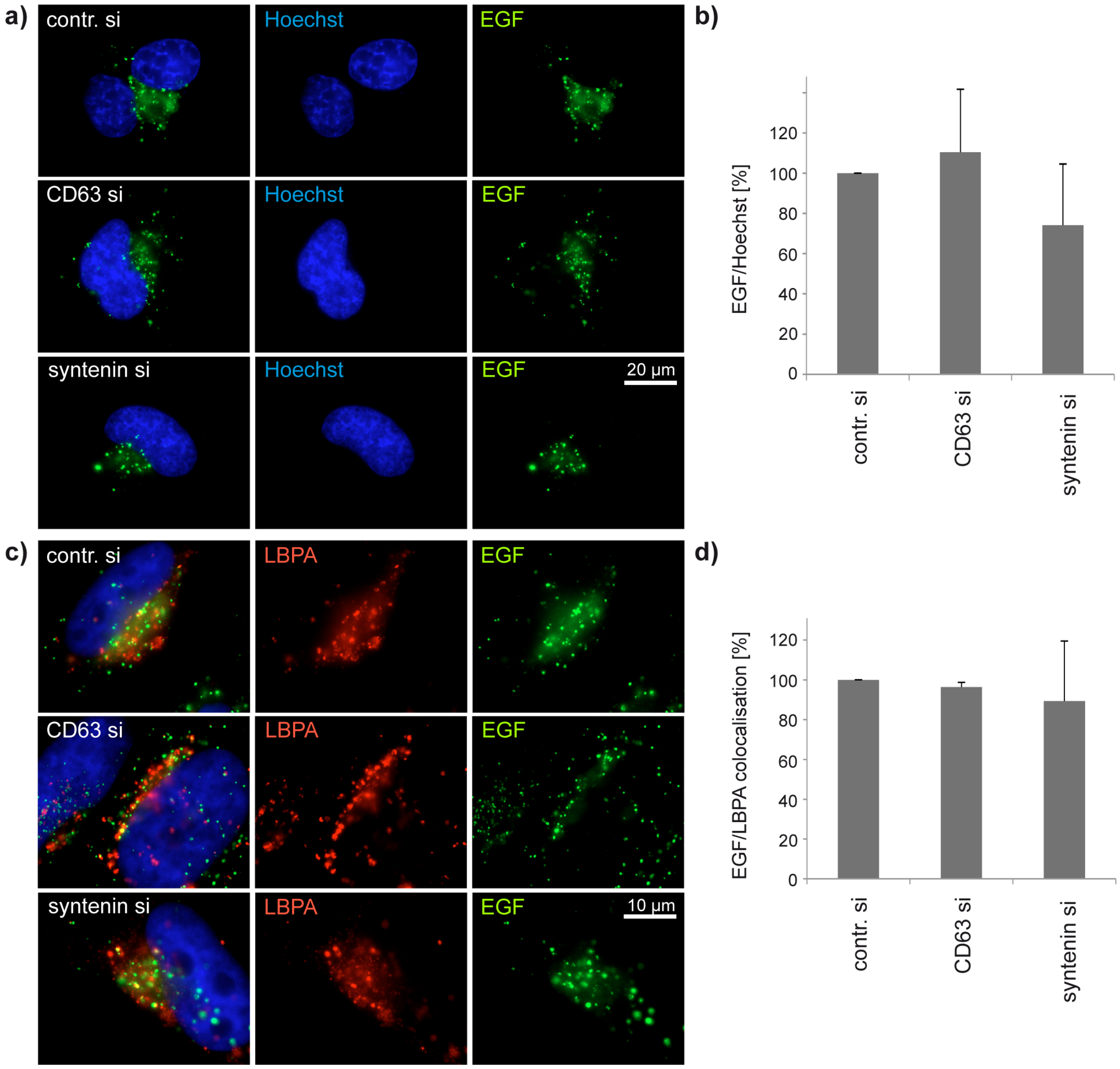

S3 Fig. CD63 and syntenin-1 specific siRNAs have no significant influence on EGF-uptake and perinuclear accumulation. (**a-d**) Hela cells were depleted of CD63 or syntenin-1 or treated with control siRNA. (**a**) EGF-Alexa Fluor 488 was added to cells for 1 hour and analysed by fluorescent microscopy after acid wash. Representative pictures show EGF (green) and nuclei (blue). (**b**) Quantification of relative of EGF staining per cell was performed by analysis of EGF-positive pixels of at least 30 images (3-5 cells per image) using ImageJ-script. (**c**) EGF was added as in (**a**), cells were immunostained with LBPA mAb (red) and colocalisation was analysed. Representative pictures show EGF (green), LBPA (red) and nuclei (blue). (**d**) Quantification of LBPA-colocalising EGF was performed by analysis of at least 12 images (3-5 cells per image) using Colocalisation Software 4.7 (Zeiss). Shown is the amount of EGF pixels relative to the total amount of LBPA pixels. Shown are the results of two independent experiments using CD63 and syntenin-1 siRNAs normalized to control siRNA treated cells (**b,d**).

						MW Score			MW ppm			SD ppm		
	Accession	Entry	Description	mW (Da)	pI (pH)	NI	4h	7h	NI	4h	7h	NI	4h	7h
HeLa 1	P03101.2	VL1_HPV16	HPV16 Major capsid protein L1	59435	7,95		722,07	1.399,76		1.774,28	3.306,23		346,96	218,52
HeLa 2	P03101.2	VL1_HPV16	HPV16 Major capsid protein L1	59435	7,95		1.411,51	2.651,65		2.054,03	3.582,00		510,08	592,75
MW ppm (1,2)	P03101.2	VL1_HPV16	HPV16 Major capsid protein L1							1.914,15	3.444,12			
HeLa 1	O00560	SDCB1_HUMAN	Syntenin 1 Syndecan binding protein 1 Melanoma differentiation associated protein 9 MDA 9	32423	7,35	2.054,00	1.114,00	1.066,00	922,00	1.727,00	2.447,00	334,75	1.310,89	1.172,89
HeLa 2	O00560	SDCB1_HUMAN	Syntenin 1 Syndecan binding protein 1 Melanoma differentiation associated protein 9 MDA 9	32423	7,35	2.040,21	3.366,01	2.769,78	1.033,90	1.374,60	2.868,35	153,22	89,49	1.162,83
MW ppm (1,2)	O00560	SDCB1_HUMAN	Syntenin 1 Syndecan binding protein 1 Melanoma differentiation associated protein 9 MDA 9						977,95	1.550,80	2.657,67			
HeLa 1	P20339	RAB5A_HUMAN	Ras related protein Rab 5A Homo sapiens Human	23643	8,25	2.507,31	3.173,17	765,94	637,21	456,39	577,39	177,66	162,25	423,04
HeLa 2	P20339	RAB5A_HUMAN	Ras related protein Rab 5A Homo sapiens Human	23643	8,25	1.532,90	1.694,38	815,54	194,47	298,19	549,81	58,52	58,79	374,76
MW ppm (1,2)	P20339	RAB5A_HUMAN	Ras related protein Rab 5A Homo sapiens Human						415,84	377,29	563,60			
HeLa 1	P61026	RAB10_HUMAN	Ras related protein Rab 10 Homo sapiens Human	22526	8,57	6.191,60	7.919,07	4.500,36	1.142,49	1.661,24	1.292,27	151,18	296,95	465,33
HeLa 2	P61026	RAB10_HUMAN	Ras related protein Rab 10 Homo sapiens Human	22526	8,57	6.419,57	6.451,02	5.734,17	1.022,18	1.249,42	1.061,37	248,55	154,07	265,18
MW ppm (1,2)	P61026	RAB10_HUMAN	Ras related protein Rab 10 Homo sapiens Human						1.082,33	1.455,33	1.176,82			
HeLa 1	P05023	AT1A1_HUMAN	Sodium potassium transporting ATPase alpha 1 chain precursor EC 3 6 3 9 Sodium pump 1 Na K	112824	5,17	5.946,90	5.020,40	3.997,18	14.475,28	16.976,67	16.495,74	949,56	841,13	1.607,21
HeLa 2	P05023	AT1A1_HUMAN	Sodium potassium transporting ATPase alpha 1 chain precursor EC 3 6 3 9 Sodium pump 1 Na K	112824	5,17	7.460,48	7.215,48	5.060,68	15.846,75	14.752,45	14.627,68	2.097,79	1.945,47	1.925,19
MW ppm (1,2)	P05023	AT1A1_HUMAN	Sodium potassium transporting ATPase alpha 1 chain precursor EC 3 6 3 9 Sodium pump 1 Na K						15.161,02	15.864,56	15.561,71			
HeLa 1	P16070	CD44_HUMAN	CD44 antigen precursor Phagocytic glycoprotein I PGP 1 HUTCH I Extracellular matrix receptor	81503	4,98	3.073,94	1.449,38	833,98	10.009,02	8.330,98	6.296,78	736,30	1.654,24	811,18
HeLa 2	P16070	CD44_HUMAN	CD44 antigen precursor Phagocytic glycoprotein I PGP 1 HUTCH I Extracellular matrix receptor	81503	4,98	3.607,45	3.652,24	1.955,03	12.367,47	12.129,12	12.054,43	1.070,16	2.392,79	1.865,93
MW ppm (1,2)	P16070	CD44_HUMAN	CD44 antigen precursor Phagocytic glycoprotein I PGP 1 HUTCH I Extracellular matrix receptor						11.188,25	10.230,05	9.175,61			
HeLa 1	P53985	MOT1_HUMAN	Monocarboxylate transporter 1 MCT 1 Solute carrier family 16 member 1 Homo sapiens Human	53922	8,72	2.079,87	1.834,62	770,21	1.997,60	2.244,48	1.714,76	274,55	425,77	348,01
HeLa 2	P53985	MOT1_HUMAN	Monocarboxylate transporter 1 MCT 1 Solute carrier family 16 member 1 Homo sapiens Human	53922	8,72	2.108,14	2.138,71	1.251,20	2.310,41	2.347,38	2.356,01	299,42	595,88	762,08
MW ppm (1,2)	P53985	MOT1_HUMAN	Monocarboxylate transporter 1 MCT 1 Solute carrier family 16 member 1 Homo sapiens Human						2.154,00	2.295,93	2.035,38			
HeLa 1	P61586	RHOA_HUMAN	Transforming protein RhoA precursor H12 Homo sapiens Human	21754	5,73	8.279,08	5.873,77	7.352,42	809,16	1.031,93	1.022,22	87,20	364,60	212,71
HeLa 2	P61586	RHOA_HUMAN	Transforming protein RhoA precursor H12 Homo sapiens Human	21754	5,73	9.716,51	11.079,98	7.499,55	1.298,45	1.184,65	1.265,24	287,41	285,63	333,90
MW ppm (1,2)	P61586	RHOA_HUMAN	Transforming protein RhoA precursor H12 Homo sapiens Human						1.053,80	1.108,29	1.143,73			
HeLa 1	P02786	TFR1_HUMAN	Transferrin receptor protein 1 TfR1 TR TfR Trfr CD71 antigen T9 p90 Homo sapiens Hu	84818	6,16	5.492,73	4.534,69	3.084,53	8.564,36	12.459,29	11.727,48	367,60	1.005,13	1.028,21
HeLa 2	P02786	TFR1_HUMAN	Transferrin receptor protein 1 TfR1 TR TfR Trfr CD71 antigen T9 p90 Homo sapiens Hu	84818	6,16	5.926,01	5.870,78	3.721,32	8.658,22	9.227,49	9.414,78	624,47	995,81	780,05
MW ppm (1,2)	P02786	TFR1_HUMAN	Transferrin receptor protein 1 TfR1 TR TfR Trfr CD71 antigen T9 p90 Homo sapiens Hu						8.611,29	10.843,39	10.571,13			

S4 Fig. Mass spectrometry analysis revealed enrichment of syntenin-1 in endosomal fractions during the time course of infection. HeLa cells were infected with HPV16 PsV for indicated time points and the amount of HPV16 L1, syntenin-1 and Rab5a protein and selected host cell factors were analysed by quantitative mass spectrometry. Rab5a is a marker of early endosomes and served as input control. Shown is the analysis of two individual qLC-MS experiments (HeLa1, HeLa2). Per experiment, three preparations (non-infected, 4h or 7h post infection) were analysed. Each preparation was analysed in five technical replicates.

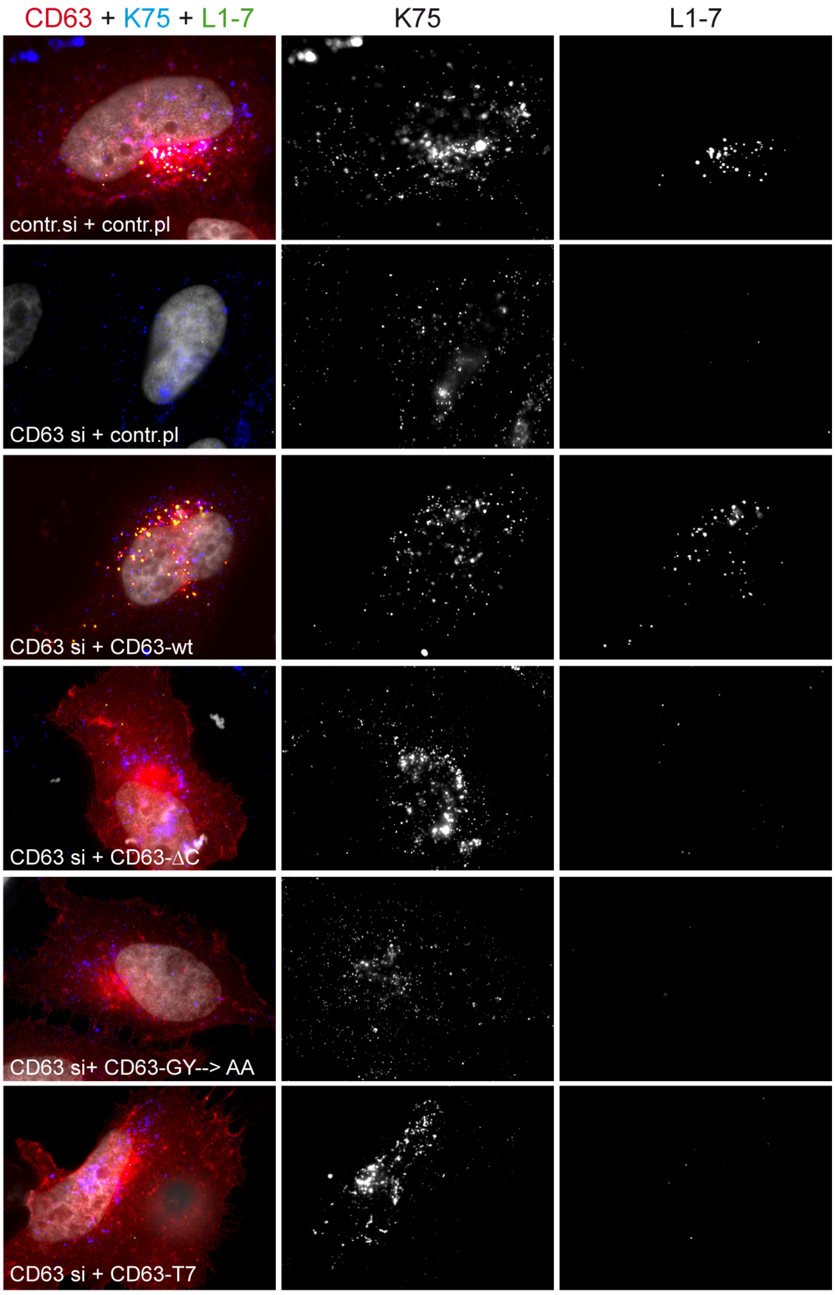

S5 Fig. Pre-disassembled PsV can be detected in cells expressing L1-7 reactivity-inhibiting mutants of CD63. Shown are representative pictures of HeLa cells treated with CD63 siRNA and transfected with CD63 wild-type or mutant plasmids. Post-uncoating processed virions were labeled with mAb 33L1-7 (green), total L1 with pAb K75 (blue) and CD63 with mAb (red).

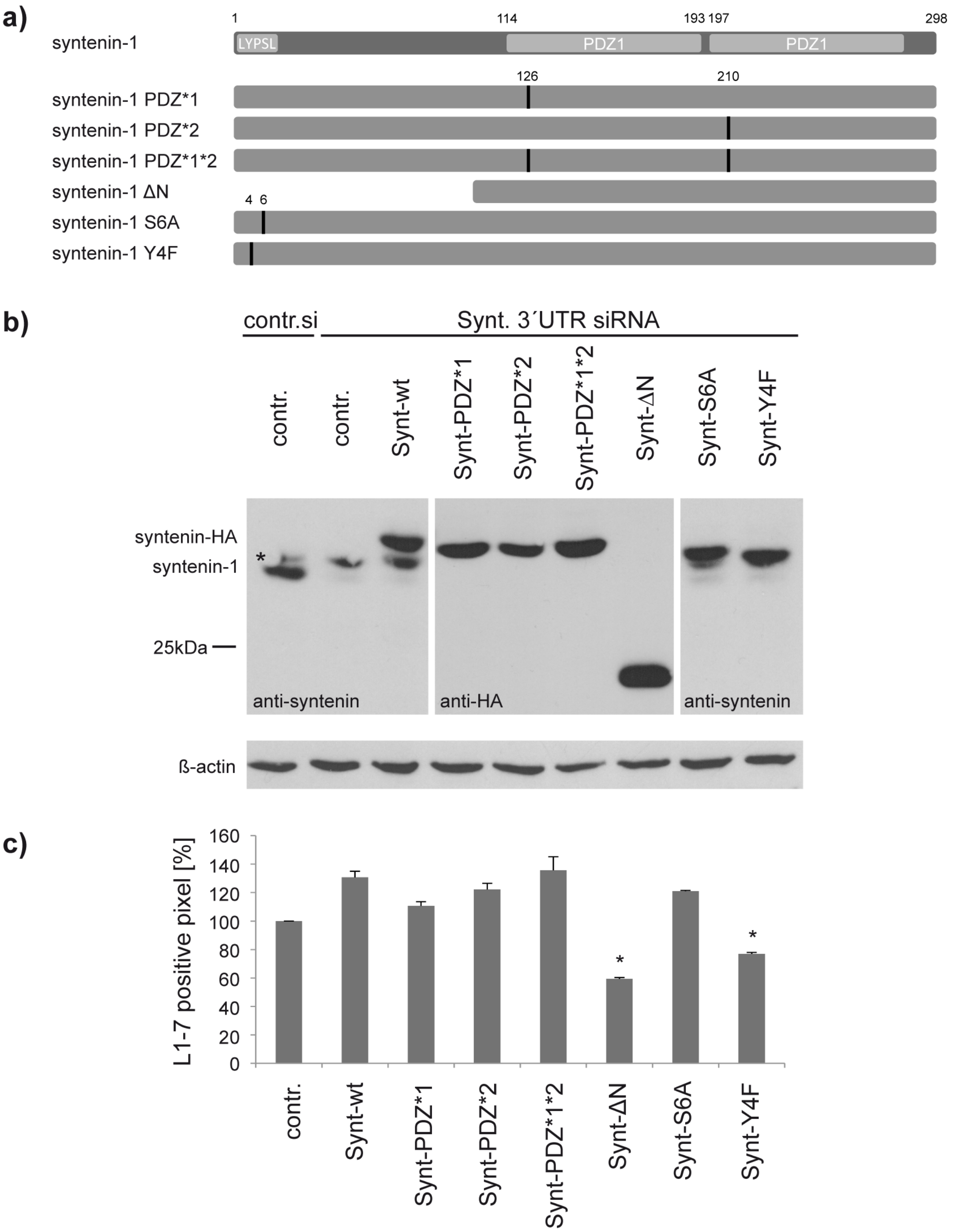

S6 Fig. Syntenin-1 mutants. (**a**) shows schematic diagram of syntenin-1 mutants. (**b**) Control or syntenin-1-depleted HeLa cells were transfected with control or syntenin-1 wild-type resp. mutant expression plasmid. Syntenin-1 knockdown of endogenous syntenin-1 and expression of syntenin-1 wild-type (synt-wt) or syntenin-1 mutants are shown in Western blot. (**c**) Syntenin-1 was overexpressed by transfection with control or syntenin-1 wild-type resp. mutant expression plasmid. L1-7 reactivity was measured as in Figs **2e,f**. *: $P < 0.05$ significant decrease compared to control.

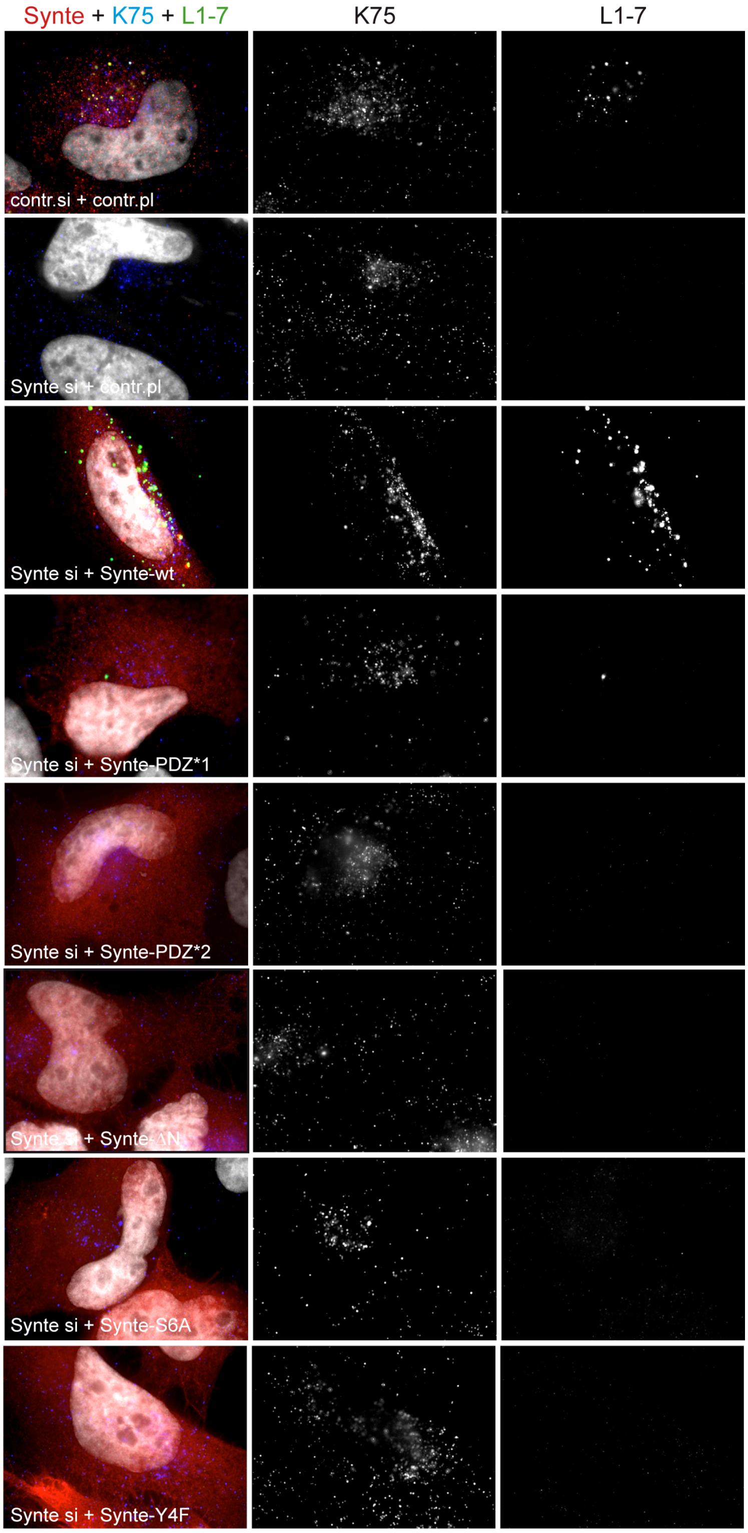

S7 Fig. Pre-disassembled PsV is detectable in cells expressing L1-7 reactivity-inhibiting syntenin-1 mutants. siRNA transfected cells were infected with HPV16 PsV after re-expression of syntenin-1 mutants. Post-uncoating virions were labeled with mAb 33L1-7 (green), total L1 with pAb K75 (blue) and syntenin-1 with mAb (red).
